# Open-Bud Duplicate Loci Are Identified as *MML10s*, Orthologs of MIXTA-Like Genes on Homologous Chromosomes of Allotetraploid Cotton

**DOI:** 10.3389/fpls.2020.00081

**Published:** 2020-02-18

**Authors:** Wei Chen, Jinbo Yao, Yan Li, Shouhong Zhu, Yan Guo, Shengtao Fang, Lanjie Zhao, Junyi Wang, Li Yuan, Youjun Lu, Yongshan Zhang

**Affiliations:** ^1^ State Key Laboratory of Cotton Biology, Institute of Cotton Research, Chinese Academy of Agricultural Sciences, Anyang, China; ^2^ School of Biological Science and Food Engineering, Anyang Institute of Technology, Anyang, China

**Keywords:** open-bud, MIXTA-like ortholog, MYB gene, map-based cloning, cotton (*Gossypium* spp.)

## Abstract

The open-bud (*ob*) mutants in cotton display abnormal flower buds with the stigma and upper anthers exposed before blooming. This characteristic is potentially useful for the efficient production of hybrid seeds. The recessive inheritance pattern of the *ob* phenotype in allotetraploid cotton is determined by duplicated recessive loci (*ob1ob1ob2ob2*). In this study, *ob1,* which is a MIXTA-like MYB gene on chromosome D13 (*MML10_Dt*), was identified by map-based cloning. In *Gossypium barbadense* (Gb) acc. 3–79, a single nucleotide polymorphism (SNP) (G/A) at the splice site of the first intron and an 8-bp deletion in the third exon of *MML10_Dt* were found, which are the causative mutations at the *ob1* loci. A 1783-bp deletion that leads to the loss of the third exon and accounts for the causal variation at the *ob2* loci was found in *MML10_At* of *Gossypium hirsutum* (Gh) acc. TM-1. The *ob* phenotype results from the combination of these two loss-of-function loci. Genotyping assays showed that the *ob1* and *ob2* loci appeared after the formation of allotetraploid cotton and were specific for Gb and Gh, respectively. All Gb lines and most Gh cultivars carry the single corresponding mutant alleles. Genome-wide transcriptome analysis showed that some of the MYB genes and genes related to cell wall biogenesis, trichome differentiation, cytokinin signal transduction, and cell division were repressed in the *ob* mutants, which may lead to suppression of petal growth. These findings should be of value for breeding superior *ob* lines in cotton.

## Introduction

Cotton (*Gossypium* spp.) is an important economic crop worldwide. The genus *Gossypium* L. is usually divided into eight diploid genome groups (A−G, and K; 2n = 2x = 26) and a single tetraploid lineage (AD1−AD7; 2n = 4x = 52) (Grover et al., 2015; Wendel and Grover, 2015; Gallagher et al., 2017). All allopolyploids in *Gossypium* were thought to share a common ancestry formed by the ancient merger and chromosome doubling of A- and D-genome ancestors (Grover et al., 2012). In tetraploid cotton, inheritance indicating duplicate loci has been found for many traits, such as yellow petal, anthocyanin pigmentation, and open bud (Harland, 1929; Endrizzi and Taylor, 1968).

Open bud (*ob*) is a flower bud mutant in *Gossypium hirsutum* (Gh) first reported by Kohel (1973). No *ob* mutant is found in diploids, but it often appears in the segregating generation of the cross between Gh (AD1) and *Gossypium barbadense* (Gb) (AD2). In the *ob* mutant, flower buds open at the tip, and the stigma and upper anthers are exposed before blooming. Genetic analysis showed that a single recessive locus (*ob1*) was responsible for the *ob* phenotype in Gh, which was subsequently mapped to Chr.18 (D13 of D subgenome) by monosomic testing (Endrizzi, 1975). Further research showed that in the allotetraploids the *ob* phenotype is due to duplicate recessive genes, and the other *ob* locus (*ob2*) is mapped to Chr.13 (A13 of A subgenome) (Endrizzi and Nelson, 1990; Endrizzi and Ray, 1991). Gh and Gb were thought to have the monomeric genotype of *Ob1Ob1ob2ob2* and *ob1ob1Ob2Ob2*, respectively (Zhang and Guo, 2001).

The *ob* phenotype was believed to have the potential for production of hybrid seeds in cotton. Thus, identification of genes responsible for the *ob* phenotype is necessary for molecular marker-assisted breeding. Qian et al. (2009) reported the rough mapping of the *ob* genes using simple sequence repeat (SSR) markers. The *ob1* and *ob2* genes were mapped to 3.4- and 2.8-centiMorgan (cM) genetic intervals on D13 and A13, respectively. In this study, we established the position of the *ob1* locus more accurately to a ~400-kb interval, characterized the underlying mutations responsible for the *ob* phenotype, and investigated the origin of the mutations. We also compared the genome-wide transcriptome between the wild type (WT) and *ob* mutants and found some key genes and pathways related to the *ob* phenotype.

## Materials and Methods

### Plant Materials and Growth Conditions

The standard genetic line in *G. hirsutum*, TM-1, and a backcrossed chromosome substitution line, CS-B18, were used in genetic mapping and gene expression analyses. CS-B18 was kindly made available by Dr. Saha of the Southern Plains Agricultural Research Center, the United States Department of Agriculture–Agricultural Research Service (USDA-ARS). CS-B18 was found to show a typical *ob* phenotype in a previous study (Saha et al., 2015). It contains a homologous pair of chromosome 18 (D13) of 3–79 (*G. barbadense*) substituting for the homologous TM-1 chromosomes (Saha et al., 2006). The F_1_ plants of the cross TM-1 × CS-B18 were self-pollinated or backcrossed with CS-B18 to produce the F_2_ or BC_1_ population. Another Gh line, YangOB, was an *ob* cultivar provided by the Hunan Institute of Cotton Sciences, China. For genotyping analysis of the *ob* gene, 26 diploid lines, 5 allotetraploid lines, 300 Gh cultivars, 533 wild Gh accessions, 214 *G. arboreum*, and 374 Gb lines were used.

The cotton plants for phenotypic investigation and DNA extraction were grown in the field at the Experimental Station of the Cotton Research Institute of the Chinese Academy of Agricultural Sciences (Anyang, China). The *ob* phenotype was scored based on visual inspection. Five buds at −10 days post-anthesis (DPA) were surveyed for each plant of the F_2_ or BC_1_ population. Petal/bract length was measured vertically from the base to the apex of petal/bract. Petal/bract width was measured horizontally at the widest position of petal/bract. The boll setting rate was calculated using the formula of (number of mature bolls/number of buds initially produced) × 100.

### DNA Extraction, SSR Amplification, and Linkage Analysis

Genomic DNA was extracted from leaves as reported in Paterson et al. (1993). The procedure for SSR analysis was performed following the method of Zhang et al. (2002). DNA bands were visualized with silver staining. MAPMAKER version 3.0b (Lander et al., 1987) was employed to construct the linkage map. The SSR primers are described in **Table S1**.

### Scanning Electron Microscopy

Samples were prepared as previously described (Wang et al., 2015). Petal samples were fixed in methanol and then serially dehydrated by ethanol. After incubation in isoamyl acetate, samples were freeze-dried, gold coated, and imaged using a Hitachi S-3000 N scanning electron microscope. Accelerating voltage was set to 1.5 kV, and magnification was set to 50.

### Sequence Comparison

The candidate gene was amplified from the cDNA of TM-1, CS-B18, and YangOB using the primers described in **Table S1** by KOD-Plus-Neo DNA polymerase (Toyobo, Shanghai, China). The polymerase chain reaction (PCR) products were subcloned into a pEASY-Blunt cloning vector (Transgen, Beijing, China), and no fewer than eight clones were sequenced for each PCR product. Sequence alignment was performed using ClustalW2 (http://www.ebi.ac.uk/Tools/msa/clustalw2/).

### RNA Extraction and Quantitative Reverse Transcription PCR

Total RNA was isolated from different tissues using the RNAprep Pure Plant Kit (Tiangen, Beijing, China). First-strand cDNA was prepared using a PrimeScript^®^ RT reagent kit with a gDNA Eraser (Takara, Dalian, China). PCR amplifications were performed using SYBR^®^ Premix Ex Taq™ (Tli RNase H Plus) (Takara, Dalian, China). Cotton *ACTIN14* (GenBank accession number: AY305733) was used as an internal control in quantitative PCR (qPCR) assays. Relative expression levels were determined using the ∆^Ct^ method. For each set of materials, three biological and two technical replicates were used. The sequences of the quantitative reverse transcription PCR (qRT-PCR) primers are presented in **Table S1**.

### Transcriptome Analysis

Petal tissue at the early developmental stage (from <4 mm buds) was used for RNA-Seq. Total RNA was isolated using an EASYspin Plus Complex Plant RNA Kit according to the manufacturer's recommendations (Aidlab, Beijing, China). Three biological replicates were performed. RNA-Seq libraries were constructed according to the user manual and sequenced to produce 150-bp paired-end reads on BGISEQ-500 platform. Clean reads were obtained by removing sequences containing adapters and poly-N runs and low-quality reads. The clean sequence tags were mapped to a TM-1 genome (JGI v1.1 annotation, https://phytozome.jgi.doe.gov). The fragments per kilobase of exon per million reads mapped (FPKM) were calculated to indicate the expression level using cufflink (Trapnell et al., 2010). Pairwise comparisons (CS-B18 versus TM-1 and YangOB versus TM-1) were conducted to find differentially expressed genes by using the R program DEGseq software with the parameters: Q-value ≤ 0.001 and log2 (fold change) ≥ 1 (Wang et al., 2010). The Gene Ontology (GO) terms and KEGG enrichment analysis were conducted using the R program phyper (FDR ≤ 0.01). Sequencing data were deposited in the NCBI SRA database (BioProject accession number: PRJNA541186).

## Results

### Phenotypic Characterization of the *ob* Mutant

**Figure 1:**
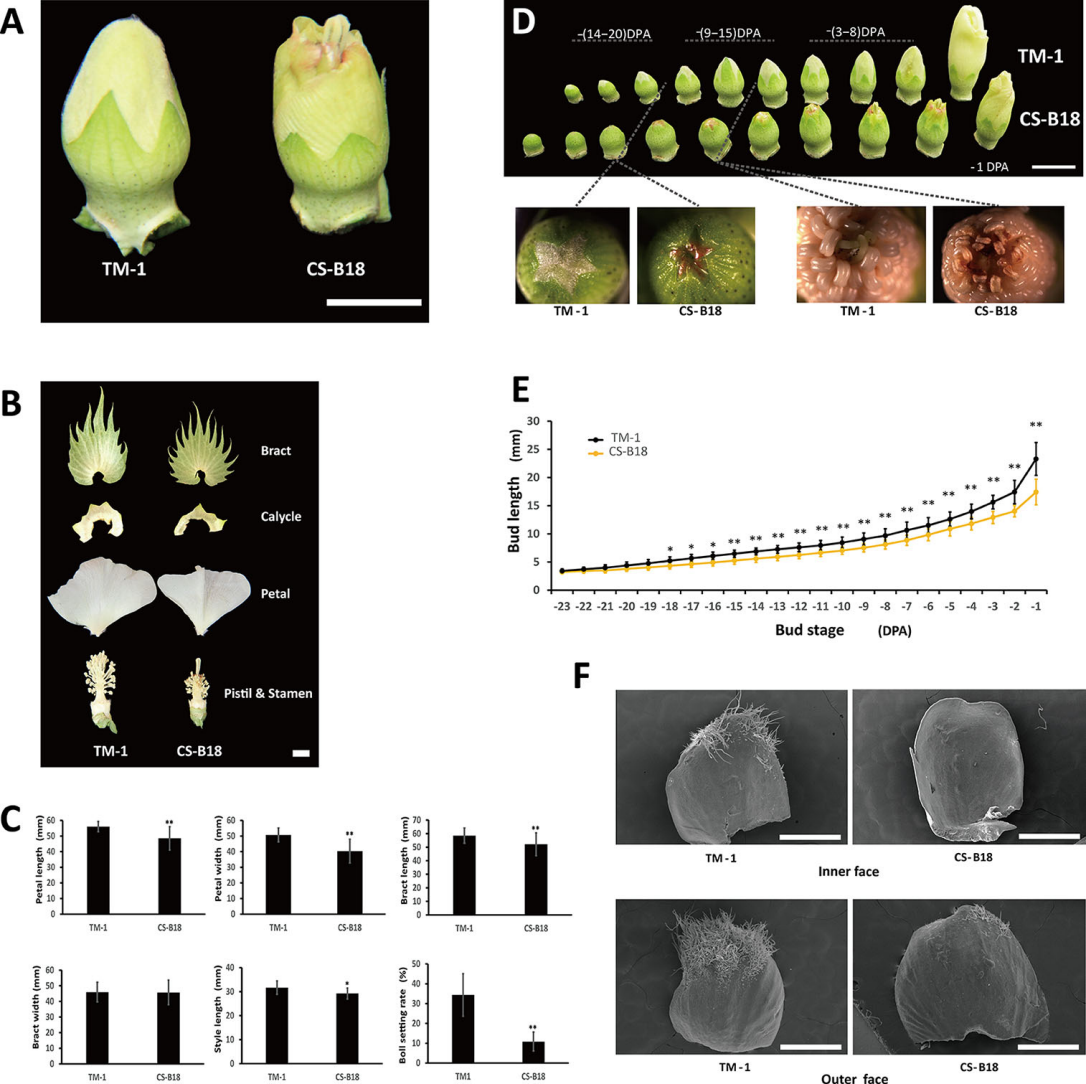
Phenotypes of wild-type (WT) and open-bud (ob) mutants. **(A)** Flower bud phenotype of TM-1 (WT) and CS-B18 (*ob*). **(B)** Different floral tissues. **(C)** Quantitative comparison of tissue size and boll setting rate between TM-1 (WT) and CS-B18 (*ob*). All values are the mean ± standard deviation (for tissue size, n = 20; for boll setting rate n = 10). **(D)** Flower bud morphology of the WT and *ob* at different developmental stages. DPA, day post-anthesis. **(E)** Summary of bud length at different developmental stage. All values are the mean ± standard deviation (n = 10). **(F)** The petals of −20 DPA-stage buds from WT and *ob* imaged using a scanning electron microscope. Statistically significant differences were revealed using Student's t-test: *, *P* < 0.05; **, *P* < 0.01. Bars: in **(A, B, D)** 1 cm; in **(F)** 1 mm.

In WT plants during flower bud development, stigma and anthers are wrapped by petals until blooming begins. In the *ob* mutant, the stigma and the developing upper anthers are exposed before anthesis (**Figure 1A**). This exposure is the consequence of shortened petal length, although length of style is also slightly shortened (**Figures 1B, C**). The *ob* mutant also displays a smaller bract and calycle, and has a shorter filament length than the WT. The *ob* phenotype could be observed as early as when the bud length has reached about 5 mm in CS-B18, and TM-1 showed significantly longer flower buds starting at −18 DPA (**Figures 1D, E**). The exposure of stigma and anthers at the early budding stage usually leads to necrosis of the upper anthers and premature desiccation of stigma, though the pollen from lower anthers seems to disperse normally. As a result the seed setting rate in the *ob* mutant is significantly lower under conditions of natural self-pollination and many more bolls are shed before maturity (**Figure 1C**). In addition, the density and numbers of trichomes on petals are dramatically reduced in the *ob* mutant, for both sides (**Figure 1F**).

### Map-Based Cloning of the *ob* Gene

**Figure 2:**
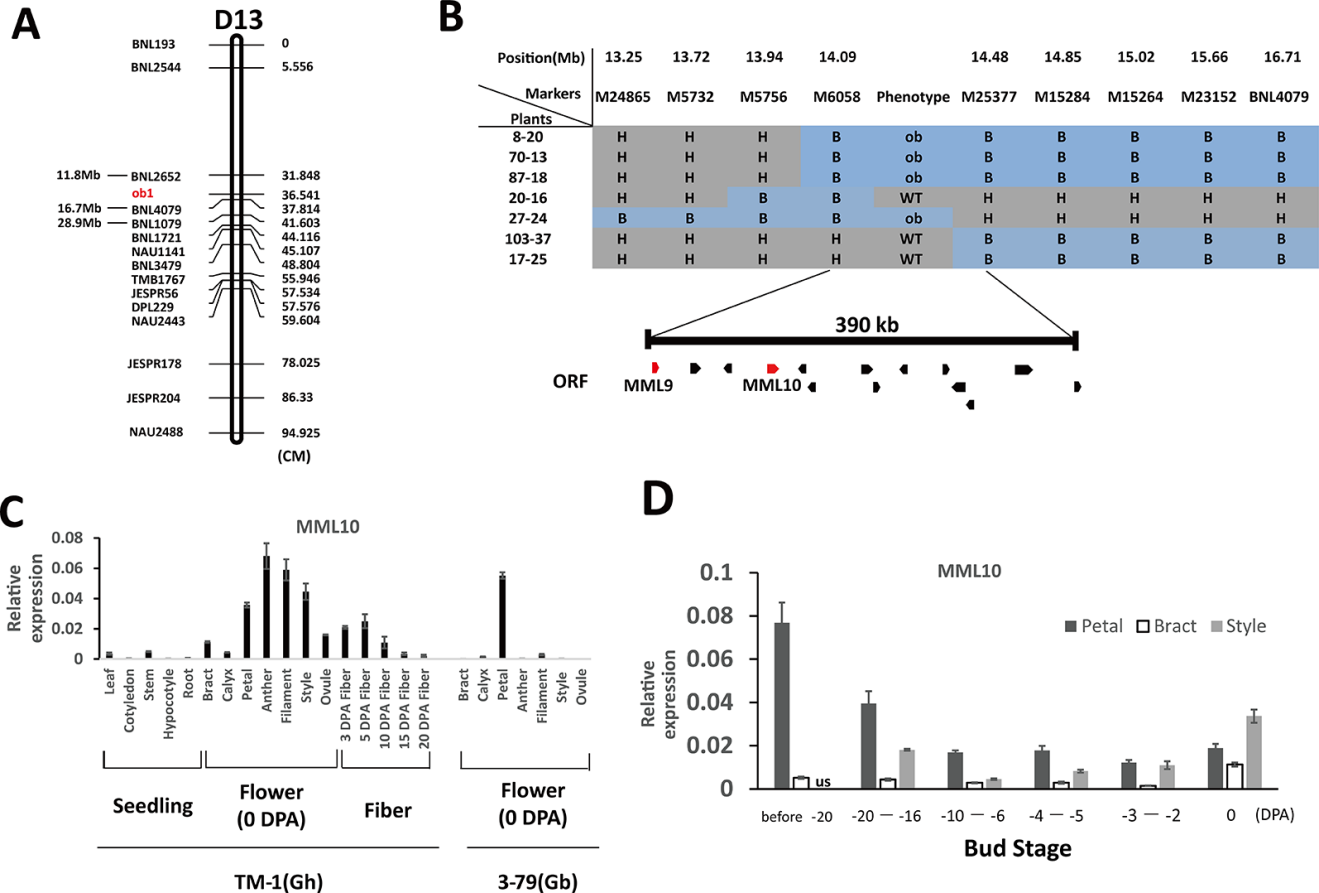
Map-based cloning of the *ob*1 gene. **(A)** Preliminary mapping of *ob*1 using 720 F_2_ plants of the cross TM-1 × CS-B18. cM, centiMorgans. **(B)** Fine mapping of *ob*1. H, heterozygous genotype; B, homozygous CS-B18 genotype; ORF, open reading frames. **(C)** Tissue-specific expression patterns in TM-1 and 3–79. Seedling tissues were gathered at the three-leaf stage. Flower tissues were gathered at anthesis. **(D)** Changes in the level of expression of MML10 at different developmental stages of buds of TM-1. us not assessed. Three biological and two technical replicates were used in qRT-PCR assay. Error bars indicate the standard deviation of three biological replicates.

In the genetic map of Qian et al. (2009), the two flanking SSR markers of *ob1*, BNL1079 and BNL2652, had the same genetic position. However, these two markers were ≈17 Mb apart with respect to their physical positions (**Figure 2A**). To reconstruct the genetic map around the *ob1* gene, in 2013 we established a primary mapping population comprising 720 F_2_ plants from the cross of TM-1 × CS-B18. Phenotypic surveys showed that segregation of the open bud phenotype corresponded to a one-locus genetic model as expected, with a 546:174 segregation ratio of WT: ob (χ^2^ = 0.107, p > 0.05). We constructed a linkage group using 15 polymorphic SSR markers on chromosome D13 between TM-1 × CS-B18 (**Figure 2A**). *ob1* was located within a 6-cM genetic interval defined by the two SSR markers BNL2652 and BNL4079, whose physical locations were 11.8 and 16.2 Mb on D13, respectively. To finely map the *ob1* gene, we used a further 4333 F_2_ and 1804 BC_1_F_1_ [TM-1 × (TM-1 × CS-B18)] plants for linkage analysis. BNL4079 and eight newly developed SSR markers were used for genotyping of these plants. Finally, the *ob1* locus was narrowed down to a ~400-kb interval between the markers M6058 and M25377 (**Figure 2B**).

According to the TM-1 reference genome sequence (Zhang et al., 2015), 14 genes are annotated in this region (**Table S2**). Out of these, two typical R2R3-MYB genes (*Gh_D13G0799* and *Gh_D13G0802*) were found, and both of them showed a high degree of similarity to members in subgroup 9 of MYBs in *Arabidopsis* and *MIXTA* (*MIXTA*-like) genes from other species (Bedon et al., 2014). In snapdragons (*Antirrhinum majus*), the *MIXTA* (*MIXTA*-like) genes have been found to control the development of petal epidermal cells (Noda et al., 1994; Baumann et al., 2007). Thus, these two *MIXTA*-like genes appear to be good candidates for the *ob1* gene. In public RNA-Seq data (Yuan et al., 2015; Zhang et al., 2015), *Gh_D13G0799* (designated as *GhMML9_Dt*) has nearly no expression in any of the surveyed tissues of TM-1, and the same was observed in 3–79 (**Table S3**). Its homoeolog, *GhMML9_At*, seemed to be preferentially expressed in ovules during the fiber initiation stage (−1 to 1 DPA). A nonsynonymous SNP and a 3-bp indel were found at the end of *MML9_Dt* between TM-1 and 3–79 (**Figure S1**). However, these mutations were located well outside the conserved MYB repeat regions (**Figure S2**), so are unlikely to affect its DNA-binding function. The other MYB gene in the interval, *Gh_D13G0802* (designated as *GhMML10_Dt*), has previously been shown, when silenced by RNAi, to result in a phenotype very similar to *ob* and a reduction in the density of trichomes on petals (Tan et al., 2016). *GhMML10_Dt* therefore appears to be the best candidate for *ob1*.

In previous RNA-Seq data, *GhMML10_At* showed the highest expression in fibers at elongating stages (5 and 10 DPA), calycle, stamen, petal, and 1–5 DPA ovules (**Table S3**). Almost no *GhMML10_Dt* was detected in any tissues except for the calycle, petal, receptacle, and leaf, all of which showed very low expression (Zhang et al., 2015). In 3–79, *GbMML10_At* was preferentially expressed in petal tissue, and it showed almost no expression in other tissues. Whereas, no expression of *GbMML10_Dt* was detected in any of the surveyed tissues (Yuan et al., 2015). Thus, *MML10_At* appears to be the major contributor to the total expression of *MML10* in both TM-1 and 3–79. In the present RNA-Seq experiment, we also found that *MML10_At* was expressed at a much higher level than *MML10_Dt* at the early stages of bud formation (< 4-mm length) in TM-1, CS-B18, and YangOB (**Table S4**). Our qRT-PCR analyses of MML10 showed a pattern of expression similar to that of previous RNA-Seq data (**Figure 2C**). In TM-1, the highest expression was found in filament, anther, style, and petal. In 3–79, it was only detected in petal tissue. For petals of TM-1 at different developmental stages of buds, *MML10* was highly expressed in the early stages of development (before about −20 DPA), but the expression declined thereafter (**Figure 2D**). Interestingly in TM-1, its expression increased again at 0 DPA; the same trend was observed for *MML10* expression in the style and was also increased in the bract at 0 DPA.

When the promotor sequences of *MML10* were aligned, a ≈800-bp region upstream of the start codon showed high conservation among Gh, Gb, *G. arboreum* and *G. raimondii* (**Figure S3**). Within this region, only one SNP (at −658 nts with predicted transcription start site set as 0) was found between TM-1 and 3–79 for *MML10_Dt*. Our RNA-Seq showed that *MML10_Dt* of TM-1 had a higher expression level than those of CS-B18 and YangOB (**Table S4**). However, the difference in expression is unlikely to be ascribed to this SNP because it is not located in any potential transcription factor (TF) binding site or core promoter element. No sequence homology was found between At (A2) and Dt (D5) copies of *MML10* upstream of this highly conserved region, although within each sub-genome group high sequence conservation was observed. It seems likely that the expression biases of At and Dt copies in both tetraploid species result from the divergence in these distal regions of their promoters.

### Identification of the Causal Mutations of the *ob* Mutant

**Figure 3:**
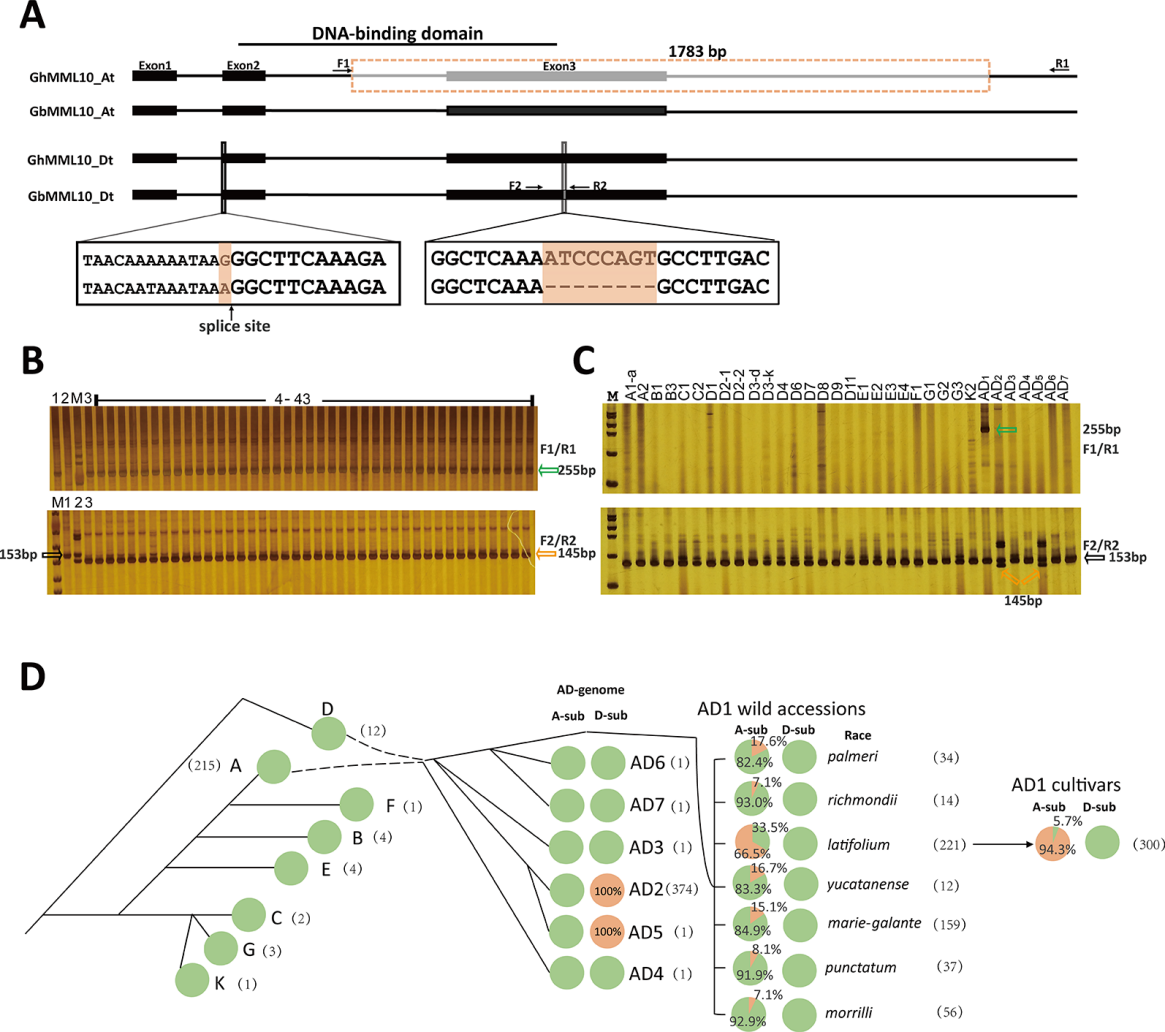
The causal variations for the *ob* phenotype in cotton. **(A)** Schematic of the exon–intron structure of the MML10 gene. Dotted boxes and lines indicate the deletion. **(B)** Genotyping of the *ob* alleles for the *ob* plants in the F2 population of TM-1 × CS-B18 using the F1/R1 and F2/R2 primer pairs. 1, TM-1; 2, 3–79; 3, CS-B18; M, marker; 4 – 43 plants displaying the *ob* phenotype. **(C)** Genotyping of the *ob* alleles for different species of *Gossypium*. Green arrows indicate the band resulting from the deletion in *GhMML10_At*. Orange arrows indicate the band resulting from the deletion in *GbMML10_Dt*. Black arrows indicate the band resulting from native *MML10*. **(D)** The genotype frequency of the *ob* gene in accessions of different species of *Gossypium*. All lines were genotyped for the 1783-bp deletion in At and the 8-bp deletion in Dt. In the pie charts, green and orange colors indicate WT and mutant genotypes, respectively. The number in parentheses is the number of accessions used for marker assay for each species. The evolutionary history is inferred from the review of Wendel and Grover (2015).

To find the underlying mutations responsible for the *ob* phenotype, genomic sequences of the *MML10* gene were retrieved from the available reference *Gossypium* genome sequences (Paterson et al., 2012; Yuan et al., 2015; Zhang et al., 2015), and cDNA sequencing was also conducted in TM-1, 3–79, and CS-B18. Sequence alignment between TM-1 and 3–79 showed that there was a 1783-bp deletion in *GhMML10_At*, which resulted in the loss of the third exon (**Figure 3A** and **Figures S4** and **S5**). The new transcript resulting from this deletion was found in our RNA-seq dataset. The truncated protein coded in the new transcript would be 87 residues long and would be missing the R3 repeat of the MYB domain, which should disrupt the normal DNA binding function of this gene (**Figure S6**). In the cDNA sequencing, only full-length transcripts of *MML10_Dt* were found in TM-1, so this homolog should generate a functional MML10 protein.

In 3–79, a SNP (G/A) at the acceptor splice site of the first intron and an 8-bp deletion in the third exon of *GbMML10_Dt* was found. In the published SNP/Indel data in Gh these two mutations in *GbMML10_Dt* are missing (Fang et al., 2017; Wang et al., 2017; Ma et al., 2018), indicating that they may be specific for Gb. In the cDNA sequencing, only full-length transcripts of *MML10_At* were found in 3–79 out of eight clones sequenced. The absence of any detected clones of *MML10_Dt* may be because its expression is much lower than *MML10_At* in floral tissues (**Table S3**). In CS-B18, the cDNA sequencing confirmed a 1-bp deletion in transcripts of *MML10_Dt* resulting from the acceptor splice site being moved forward one base because of the SNP (**Figure S5**). The truncated protein (52 residues in length) that would be produced by *GbMML10_Dt* loses both repeats of the MYB domain and any downstream activation domains so should be completely non-functional.

Thus, the mutations in *GhMML10_At* and *GbMML10_Dt* both appear to lead to loss-of-function, which fits the expected genetic model of the *ob* phenotype. When screened using PCR makers based on the mutations in *MML10*, all *ob* plants in the F_2_ population of TM-1 × CS-B18 displayed the mutant genotypes in both their At and Dt homoeologs of *MML10* (**Figure 3B**). Thus the two allotetraploid cotton (TM-1 and 3–79) each contain only one functional copy of the *MML10* gene but on opposite sub-genomes, and the *ob* phenotype results from the combination of these loss-of-function loci in the segregating generation. Interestingly, the sequence of *MML10_Dt* of YangOB corresponded exactly to that of 3–79, which implies that some apparently “spontaneous” *ob* mutants discovered in Gh are probably the result of an earlier introgression of Gb chromosome segments during breeding or through outcrossing.

We screened 26 lines representing diploid and seven allotetraploid species using markers based on the deletions to explore the emergence of these mutations in *MML10* (**Figure 3C** and **Table S5**). The 1783-bp deletion in *MML10_At* was not found in these lines except for Gh. Further screening in 214 *G. arboreum* lines indicated that this allele was also missing in the A2 genome (**Table S6**). It seems that this mutation occurred only after the formation of allotetraploid Gh species. The 8-bp deletion in *MML10_D*t was only found in *G. darwinii* (AD5) besides Gb. *G*. *darwinii* is believed to be a sister-species (or variation) of Gb (Wendel and Percy, 1990), so it is not surprising that this mutation was found in it.

Further screening showed that 283 out of 300 Gh cultivars contained the 1783-bp deletion in *GhMML10_At* (**Figure 3D** and **Table S7**). To trace the origin of this variant, we screened 533 wild Gh accessions with the marker (**Figure 3D** and **Table S8**). Out of seven geographic races, *latifolium* had the highest ratio of mutation, while all of the other races displayed relatively low ones. This finding fits the prevailing view that most of modern Gh cultivars originated from the race *latifolium* (Niles and Feaster, 1984; Meredith, 1991).

When screened with the PCR marker based on the 8-bp deletion in *GbMML10_Dt*, all Gh cultivars and wild accessions displayed the WT genotype except for one line, marie-galante 85B (from the line TX-1771). This line has very similar morphological characteristics to those of Gb, so that the mutation present in it seems to be the consequence of contamination from Gb during the maintenance in the germplasm collection. For Gb, the 374 lines assayed all contained the 8-bp deletion in *GbMML10_Dt* (**Figure 3D** and **Table S9**). The splice-site mutation was also found in these lines when screened using the corresponding SNP genotyping marker. It seems that these two mutations occurred simultaneously in Gb and early in the evolution of this species. No Gb line was found to have 1783-bp deletion in *MML10_At*, so this must have occurred after the divergence of the Gh and Gb lineages.

In summary, the mutations in *MML10* are specific for the AD1- and AD2 (AD5)-genomes and they only occurred after the formation of these two allotetraploid species. This finding is logical, considering that these mutations show a negative effect on petal development and seed set. In the allotetraploid species the functional copy on the other sub-genome masks any adverse effects; thus, the species can maintain a normal floral phenotype.

### Comparison of Genome-Wide Transcriptome Between WT and *ob* Mutants

**Figure 4:**
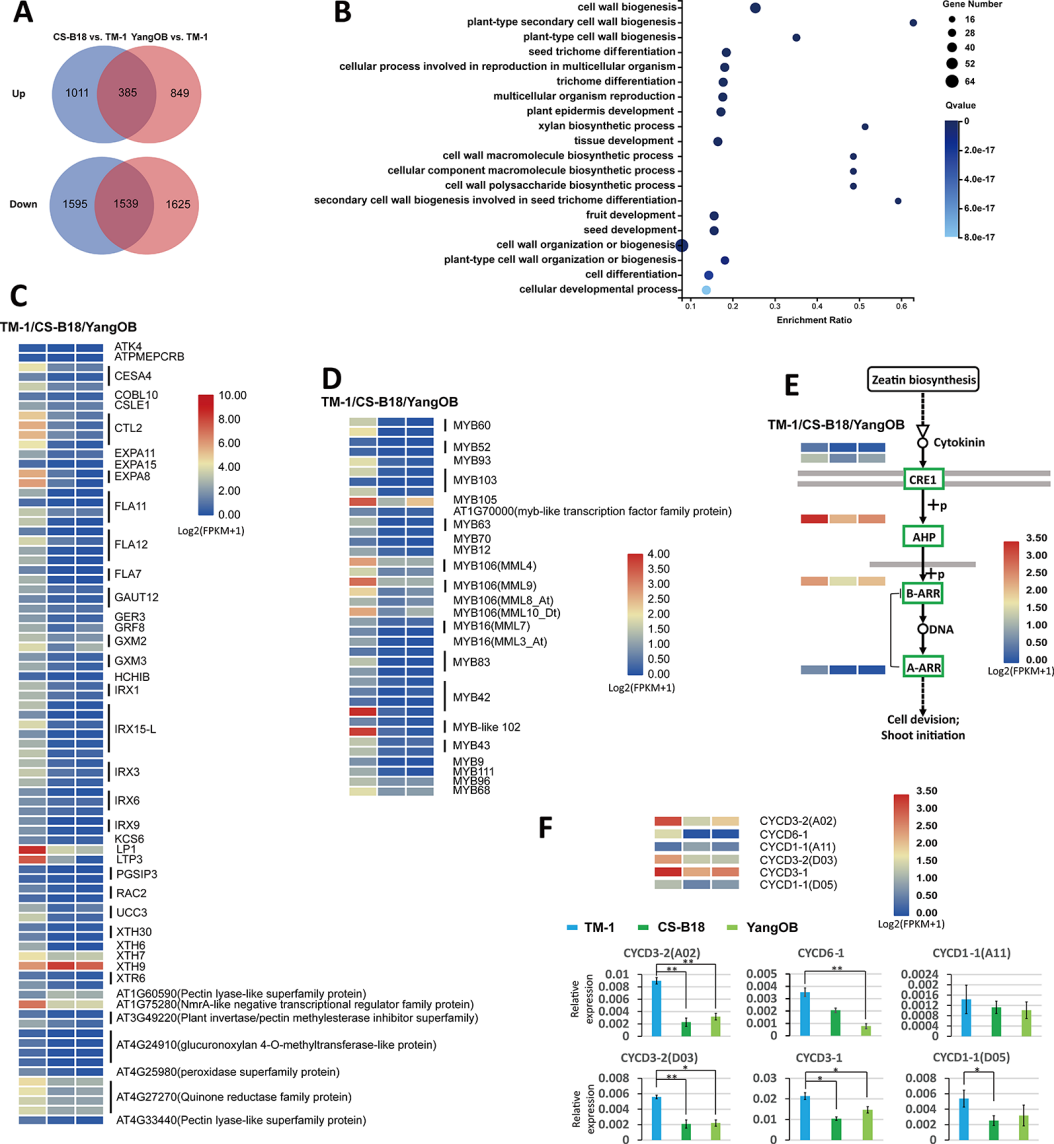
Comparison of genome-wide transcriptome between WT and *ob* mutants using RNA-Seq. **(A)** Venn diagram showing the number of differentially expressed genes. **(B)** The top 20 GO terms with the smallest Q values in GO enrichment analysis for common differentially expressed genes found in both comparisons of CS-B18 vs. TM-1 and YangOB vs. TM-1. Enrichment ratio is calculated using the formula of (number of differentially expressed genes/number of all genes for a given GO term). **(C)** Heatmap visualization of expression values (log_2_^(FPKM^^+^^1)^) of the 81 differentially expressed genes in the top 20 GO terms with the smallest Q values. The names of the homologous genes in *Arabidopsis* are given on the right. For each gene, the results of three biological replicates were averaged. **(D)** Expression heatmap visualizations of 38 MYBs. The names of homologous genes in *Arabidopsis* are indicated on the right. **(E)** Cytokinin signal transduction pathway (defined by KEGG database). Green boxes indicate the down-regulated genes in the *ob* mutants. The expression heatmaps of these genes are shown beside the gene names. **(F)** Expression heatmap visualizations and qRT-PCR analysis for six CYCD genes. Statistically significant differences were revealed using Student's t-test: *, *P* < 0.05; **, *P* < 0.01.

To analyze the transcriptional changes between WT and *ob* mutant plants, we carried out RNA-Seq analyses for the petals of <4-mm buds (before −20 DPA stage) of TM-1, CS-B18, and YangOB. A total of 1924 common genes showed differential expression between TM-1 and the *ob* mutants (**Table S10**), and most of them (1539) were down-regulated (**Figure 4A**). GO enrichment analyses of these common genes revealed that those related to the biological processes of cell wall biogenesis and trichome differentiation were significantly overrepresented (**Figures 4B, C**). In the top 20 significantly enriched GO terms, nearly all of the genes were down-regulated in *ob* mutants (**Table S10**). These genes comprise members of the alpha-expansin gene family (EXPAs), fasciclin-like arabinogalactan proteins (FLAs), irregular xylem genes (IRXs), and xyloglucan endotransglucosylase/hydrolase genes (XTHs).

There are 15 and 129 TFs up- and down-regulated out of all the common differentially expressed genes (**Table S10**). MYBs, AP2-EREBPs (APETALA2 and ethylene-responsive element binding proteins), and NACs (NAM, ATAF1/2, and CUC2) were the three categories with the highest proportion in these TFs. All these MYBs were down-regulated (**Figure 4D**). In addition to *MML10*, five other MIXTA-like MYBs (*MML3*, *MML4*, *MML7*, *MML8*, and *MML9*) were also found to be down-regulated in the *ob* mutants, and some of them have been shown to regulate fiber (or trichome) initiation or development in previous studies (Walford et al., 2011; Wu et al., 2018). Several of AP2/EREBPs factors, such as *AINTEGUMENTA* (*ANT*) and *AINTEGUMENTA-LIKE* 6 (*AIL6*), have been implicated in regulating petal cell proliferation in *Arabidopsis* (Mizukami and Fischer, 2000; Krizek, 2015; Huang and Irish, 2016).

The KEGG pathway analysis and qRT-PCR showed that genes mediating all steps of the cytokinin signal transduction pathway were down-regulated in CS-B18 (**Figure 4E**, **Figure S7**, **Table S11**). However, for the other kinds of hormones, no such consistent trend was found, although some nodes of their signal transduction pathways contained differentially expressed genes (**Figure S8**). Cytokinins are a class of plant growth substances that promote cell division, or cytokinesis. When five types of cell cycle marker genes (*CYCA*, *CYCD*, *CDKA*, *CDKB*, and *Histone H4*) were checked, only six *CYCD* genes were found to be differentially expressed between TM-1 and *ob* mutants, and five of them were down-regulated in CS-B18 (**Figure 4F**, **Table S11**). The *ob* mutant plants all show smaller petals compared to those of WT, but partial silencing of *MML10* did not appear to alter petal epidermal cell size in a previous study (Tan et al., 2016). Thus, it is possible that cell division in the petals is inhibited in the mutants through modulation of signal transduction by phytohormones.

## Discussion

A generally accepted perspective for the origin of the allopolyploid cottons is that they were generated by hybridization of an A-genome diploid (as female) with a D-genome diploid during the Pleistocene (1–2 mya) (Wendel and Grover, 2015). The actual parents of the allopolyploids are thought to be extinct. *G. raimondii* (D5) was found to be the closest living relative of the D-genome ancestor, while the two extant A-genome species (*G. herbaceum*, A1; *G. arboreum*, A2) are genealogically equidistant from the A-genome of all of the extant allopolyploids (Endrizzi et al., 1985). The results of Grover et al. (2012) supported a single origin of polyploidy in cotton. The functional consequence of allopolyploid formation was the duplication of all nuclear genes, which would lead to significant genomic change. A possible outcome of gene doubling is that one member of the duplicated gene pair could become silenced or mutated to a pseudogene (Lynch and Conery, 2000; Wendel, 2000). This is the case for the mutant *ob* genes identified in the present study. In both Gh and Gb, a higher level of expression was found for the copy on the At sub-genome. Many other duplicated MML genes generated through allopolyploidy also displayed sub-genome expression biases. For example, only a single copy (At or Dt) of *MML4*, *MML8*, and *MML9* was found to be highly expressed in ovules, and the other copy was completely silenced (Zhang et al., 2015). In addition, the mutations of *MML10_At* and *MML10_Dt* make these two copies into non-functional genes in Gh and Gb, respectively. Interestingly, these harmful mutations are maintained in modern cultivars for both species. These results imply that a possible advantage of gene duplication is the retention of some “harmful” mutations by the mitigation of purifying selection, while the mutations may afford an opportunity for artificial selection and evolution of a new function. The duplicate locus inheritance in allopolyploids cotton for many traits may be a reflection of this mechanism.

MIXTA (MIXTA-like) genes in many species have been found to regulate the shape of the epidermal cells of petals and other tissues (Noda et al., 1994; Baumann et al., 2007; Oshima et al., 2013; Huang and Irish, 2016). In cotton, two other MML genes (*MML3*, *MML4*) have been demonstrated to be the regulators of cotton fiber (a seed trichome) initiation (Walford et al., 2011; Wan et al., 2016; Wu et al., 2018). The present study on the *ob* gene confirmed the results of Tan et al. (2016) that showed that *MML10* was important for the regulation of petal trichomes in cotton. In addition, as the size of the petals was reduced in *ob* mutants this process may be just as important in causing the exposure of the stigma and upper anthers. The transcriptome analyses using RNA-Seq showed that most of the differentially expressed TFs and genes that had roles in cell wall biogenesis were down-regulated in the *ob* mutants. Thus, the *ob* gene seems to be an important upstream regulator of petal morphogenesis. Actually, in *Arabidopsis* two MYB genes, *AtMYB59* and *DUO1*, have been proven to regulate of the cell cycle (division) (Brownfield et al., 2009; Cominelli and Tonelli, 2009; Mu et al., 2009).

The utilization of the ob phenotype is thought to have the potential for cost-saving in the production of hybrid seeds because the exposure of stigma before blooming and the low level of self-fertilization obviates the need for emasculation and perianth removal. Open-bud lines, such as YangOB, have been developed by traditional breeding methods without marker-assisted selection (MAS) in China, but the low efficiency in transferring the *ob* alleles is a big obstacle in breeding programs. In practice, self-pollination and progeny testing are needed to identify the expected genotype after each backcrossing in traditional breeding. In the present study, perfect molecular markers were developed based on the coding sequences of the *ob* genes, so no genetic recombination would occur during the MAS procedure. These markers are superior to random DNA markers flanking the *ob* genes, because genetic recombination can break genetic linkage between the latter and the *ob* alleles.

## Data Availability Statement

The datasets generated for this study can be found in the BioProject accession number: PRJNA541186.

## Author Contributions

WC, JY, and YZ designed the research, performed most of experiments, and analyzed the data. YaL, YG, SZ, SF, LZ, JW, LY, and YoL performed part of the experiments. WC and YZ interpreted the results and wrote the manuscript. All of the authors have read and approved the final version of the manuscript.

## Funding

This research was funded by the National Natural Science Foundation of China (31621005).

## Conflict of Interest

The authors declare that the research was conducted in the absence of any commercial or financial relationships that could be construed as a potential conflict of interest.
