# Supplementary material for: Open-Bud Duplicate Loci Are Identified as MML10s, Orthologs of MIXTA-Like Genes on Homologous Chromosomes of Allotetraploid Cotton
Source: Front Plant Sci. 2020 Feb 18;11:81. doi: 10.3389/fpls.2020.00081 (PMC7040098; doi:10.3389/fpls.2020.00081)
Supplement: Supplementary file 1 [file DataSheet_1.zip › Figure S1.pdf]

Figure S1 Alignment of coding sequences of *MML9*.

```
         10        20        30        40        50        60        70        80        90       100
....|....|....|....|....|....|....|....|....|....|....|....|....|....|....|....|....|....|....|....|
ATGGGACGGTCTCCATGCTGTGATAAGGTGGGGTTGAAGAAAGGGCCATGGACTCCCGAAGAAGATCAAAAGCTCTTGGCTTACATTGAGCAACATGGCC GhMML9_At
ATGGGACGGTCTCCATGCTGTGATAAGGTGGGGTTGAAGAAAGGGCCATGGACTCCCGAAGAAGATCAAAAGCTCTTGGCTTACATTGAGCAACATGGCC GbMML9_At
ATGGGACGGTCTCCATGCTGTGATAAGGTGGGGTTGAAGAAAGGGCCATGGACTCCCGAAGAAGATCAAAAGCTCTTGGCTTACATTGAGCAACATGGCC GhMML9_Dt
ATGGGACGGTCTCCATGCTGTGATAAGGTGGGGTTGAAGAAAGGGCCATGGACTCCCGAAGAAGATCAAAAGCTCTTGGCTTACATTGAGCAACATGGCC GbMML9_Dt
Start codon

        110       120       130       140       150       160       170       180       190       200
....|....|....|....|....|....|....|....|....|....|....|....|....|....|....|....|....|....|....|....|
ATGGAAGCTGGCGTGCTTTGCCTGCCAAAGCTGGGCTTCAAAGATGTGGCAAGAGTTGCAGACTTAGGTGGATTAACTACTTAAGACCTGATATCAAAAG GhMML9_At
ATGGAAGCTGGCGTGCTTTGCCTGCCAAAGCTGGGCTTCAAAGATGTGGCAAGAGTTGCAGACTTAGGTGGATTAACTACTTAAGACCTGATATCAAAAG GbMML9_At
ATGGAAGCTGGCGTGCTTTGCCTGCAAAAGCTGGGCTTCAAAGATGTGGCAAGAGTTGCAGACTTAGGTGGATTAACTACTTAAGACCTGATATCAAAAG GhMML9_Dt
ATGGAAGCTGGCGTGCTTTGCCTGCAAAAGCTGGGCTTCAAAGATGTGGCAAGAGTTGCAGACTTAGGTGGATTAACTACTTAAGACCTGATATCAAAAG GbMML9_Dt

        210       220       230       240       250       260       270       280       290       300
....|....|....|....|....|....|....|....|....|....|....|....|....|....|....|....|....|....|....|....|
AGGAAAATTCAGTTTACAAGAAGAACAGACCATCATTCAACTCCATGCCCTTCTCGGAAACAGGTGGTCGGGTATTGCAGCTCATTTACCAAAAAGAACA GhMML9_At
AGGAAAATTCAGTTTACAAGAAGAACAGACCATCATTCAACTCCATGCCCTTCTCGGAAACAGGTGGTCGGGTATTGCAGCTCATTTACCAAAAAGAACA GbMML9_At
AGGAAAATTCAGTTTACAAGAAGAGCAGACCATCATTCAACTCCATGCCCTCCTCGGAAATAGGTGGTCGGGTATCGCTGCTCATTTACCAAAAAGAACA GhMML9_Dt
AGGAAAATTCAGTTTACAAGAAGAGCAGACCATCATTCAACTCCATGCCCTCCTCGGAAATAGGTGGTCGGGTATCGCTGCTCATTTACCAAAAAGAACA GbMML9_Dt
```

```
        310       320       330       340       350       360       370       380       390       400
....|....|....|....|....|....|....|....|....|....|....|....|....|....|....|....|....|....|....|....|
GACAATGAGATTAAGAACTACTGGAATACACATCTAAAGAAAAGATTGAACAAGATGGGGATCGATCCCGTGACGCAGAAGCCTAAAACTAATGCCCTCG GhMML9_At
GACAATGAGATTAAGAACTACTGGAATACACATCTAAAGAAAAGATTGAACAAGATGGGGATCGATCCCGTGACGCAGAAGCCTAAAACTAATGCCCTCG GbMML9_At
GACAATGAGATTAAGAACTACTGGAATACACATCTAAAGAAAAGATTGAACAAGATGGGGATCGATCCCGTGACCCACAAGCCTAAAACTAATGCCCTCG GhMML9_Dt
GACAATGAGATTAAGAACTACTGGAATACACATCTAAAGAAAAGATTGAACAAGATGGGGATCGATCCCGTGACCCACAAGCCTAAAACTAATGCCCTCG GbMML9_Dt

        410       420       430       440       450       460       470       480       490       500
....|....|....|....|....|....|....|....|....|....|....|....|....|....|....|....|....|....|....|....|
GGTCCGCAACCGGTAACCCCAAGGATGCAGCTACCCTCAGCCATATGGCTCAATGGGAGAGTGCTCGGTTAGAAGCCGAAGCCAGGCTGGTAAGAGAGTC GhMML9_At
GGTCCGCAACCGGTAACCCCAAGGATGCAGCTACCCTCAGCCATATGGCTCAATGGGAGAGTGCTCGGTTAGAAGCCGAAGCCAGGCTGGTAAGAGAGTC GbMML9_At
GGTCCGCAACCGGTAACCCCAAGGATGCAGCTACCCTCAGCCATATGGCTCAATGGGAGAGTGCTCGGTTAGAAGCCGAAGCCAGGCTGGTTAGAGAGTC GhMML9_Dt
GGTCCGCAACCGGTAACCCCAAGGATGCAGCTACCCTCAGCCATATGGCTCAATGGGAGAGTGCTCGGTTAGAAGCCGAAGCCAGGCTGGTTAGAGAGTC GbMML9_Dt

        510       520       530       540       550       560       570       580       590       600
....|....|....|....|....|....|....|....|....|....|....|....|....|....|....|....|....|....|....|....|
GAAATTCGTTGGCTCCTCCTCTTCCTCTTCTTCTTCTTCTTCTTCCTCGGCTCCACAGCACACTAGCAACACTGTCATGACTCCGCCGGCGACTAGGCCA GhMML9_At
GAAATTCGTTGGCTCCTCCTCTTCCTCTTCTTCTTCTTCTTCTTCCTCGGCTCCACAGCACACTAGCAACACT--------------------------- GbMML9_At
GAAACTCGTTCGCTTCTCCTCTTCC---TCCTCTTCTTCTTCTTCCTCGGCTCCACAGCACACTAGCAATCCTGTCATGACTCCGCCGGCGACTAGGCCA GhMML9_Dt
GAAACTCGTTCGCTTCTCCTCTTCC---TCCTCTTCTTCTTCTTCCTCGGCTCCACAGCACACTAGCAATCCTGTCATGACTCCGCCGGCGACTAGGCCA GbMML9_Dt

        610       620       630       640       650       660       670       680       690       700
....|....|....|....|....|....|....|....|....|....|....|....|....|....|....|....|....|....|....|....|
CAATGCCTCGATGTGCTCAAAGCTTGGCAAGGTTTAGTCACCGGATTGTTCACTTTCAATAACACAACTGACAACCTCCAGTCTCCGACGTCAACGTTGA GhMML9_At
---------------------------------------------------------------------------------TCTCCGACGTCAACGTTGA GbMML9_At
CAATGCCTCGATGTGCTCAAAGCTTGGCAAGGTTTAGTGACCGGATTGTTCACTTTCAATAATACTACTGACAACCTCCAGTCTCCGACGTCAACGTTGA GhMML9_Dt
```

```
CAATGCCTCGATGTGCTCAAAGCTTGGCAAGGTTTAGTGACCGGATTGTTCACTTTCAATAATACTACTGACAACCTCCAGTCTCCGACGTCAACGTTGA GbMML9_Dt

              710       720       730       740       750       760       770       780       790       800
         ....|....|....|....|....|....|....|....|....|....|....|....|....|....|....|....|....|....|....|....|
ACTTCGTGGAGAACACCAACACGTTGGCTAATGGATTAATCAATGAAAACTCCATGGAGTTGCATGAAATGGGTGCATGGTTTCGTCAAGATTCTTCATA GhMML9_At
ACTTCGTGGAGAACACCAACACGTTGGCTAATGGATTAATCAATGAAAACTCCATGGAGTTGCATGAAATGGGTGCATGGTTTCGTCAAGATTCTTCATA GbMML9_At
ACTTCGTGGAGAACACCAACACGTTGGCTAATGGATTAATCAATGACAACTCCATGGAGTTGCATGAAATGGGTGCATGGTTTCGTCAAGATTCTTCATA GhMML9_Dt
ACTTCGTGGAGAACACCAACACGTTGGCTAATGGATTAATCAATGACAACTCCATGGAGTTGCATGAAATGGGTGCATGGTTTCGTCAAGATTCTTCATA GbMML9_Dt

              810       820       830       840       850       860       870       880       890       900
         ....|....|....|....|....|....|....|....|....|....|....|....|....|....|....|....|....|....|....|....|
CAGGGCAGTAGAGAACATGAACATGGAAGATTATTCGGATATGATGGTCTGGGAATCTGGGGATCATCAGCAGTGGTCATCAATGGCGGCGCCAGCTGAA GhMML9_At
CAGGGCAGTAGAGAACATGAACATGGAAGATTATTCGGATATGATGGTCTGGGAATCTGGGGATCATCAGCAGTGGTCATCAATGGCGGCGCCAGCTGAA GbMML9_At
CAGGGCAGTAGAGAACATGAACATGGAAGATTATTCGGATATGATGGTTTGGGAATCTGGGGATCATCAGCAGTGTTCATCAATGGCGGCGCCAGCTGAA GhMML9_Dt
CAGGGCAGTAGAGAACATGAACATGGAAGATTATTCGGATATGATGGTTTGGGAATCTGGGGATCATCAGCAGTGTTCATCAATGGCGGCGCCAGCTGAA GbMML9_Dt

              910       920       930       940       950       960       970       980       990       1000
         ....|....|....|....|....|....|....|....|....|....|....|....|....|....|....|....|....|....|....|....|
AACTTGAACGAAACAAGCTATG---GTAATAGTAGTAGTAGTAGTAGTAGTTTAGAGGAGAATAGGAATTACTGGAATAACATCCTTAATTTGGTAAGTT GhMML9_At
AACTTGAACGAAACAAGCTATG---GTAATAGTAGTAGTAGTAGTAGTAGTTTAGAGGAGAATAGGAATTACTGGAATAACATCCTTAATTTGGTAAGTT GbMML9_At
AACTTGAACGAAACAAGCTATGGTAGTAGTAGTAGTAGTAGTAGTAGTAGTTTAGAGGAGAATAGGAATTACTGGAATAACATCCTTAATTTGGTAAGTT GhMML9_Dt
AACTTGAACGAAACAAGCTATG---GTAATAGTAGTAGTAGTAGTAGTAGTTTAGAGGAGAATAGGAATTACTGGAATAACATCCTTAATTTGGTAAGTT GbMML9_Dt
```

variations between Gh and Gb

..	
AA	*GhMML9_At*
AA	*GbMML9_At*
AA	*GhMML9_Dt*
AA	*GbMML9_Dt*
