# Supplementary material for: Open-Bud Duplicate Loci Are Identified as MML10s, Orthologs of MIXTA-Like Genes on Homologous Chromosomes of Allotetraploid Cotton
Source: Front Plant Sci. 2020 Feb 18;11:81. doi: 10.3389/fpls.2020.00081 (PMC7040098; doi:10.3389/fpls.2020.00081)
Supplement: Supplementary file 1 [file DataSheet_1.zip › Figure S2.pdf]

Figure S2 Alignment of the protein sequences of *MML9_Dt*. Letters with yellow background: DNA-binding motif.

```
         10        20        30        40        50        60        70        80        90       100
....|....|....|....|....|....|....|....|....|....|....|....|....|....|....|....|....|....|....|....|
MGRSPCCDKVGLKKGPWTPEEDQKLLAYIEQHGHGSWRALPAKAGLQRCGKSCRLRWINYLRPDIKRGKFSLQEEQTIIQLHALLGNRWSGIAAHLPKRT GbMML9_Dt(Hai7124)
MGRSPCCDKVGLKKGPWTPEEDQKLLAYIEQHGHGSWRALPAKAGLQRCGKSCRLRWINYLRPDIKRGKFSLQEEQTIIQLHALLGNRWSGIAAHLPKRT GbMML9_Dt(3-79)
MGRSPCCDKVGLKKGPWTPEEDQKLLAYIEQHGHGSWRALPAKAGLQRCGKSCRLRWINYLRPDIKRGKFSLQEEQTIIQLHALLGNRWSGIAAHLPKRT GhMML9_Dt(TM-1_ZJU)
MGRSPCCDKVGLKKGPWTPEEDQKLLAYIEQHGHGSWRALPAKAGLQRCGKSCRLRWINYLRPDIKRGKFSLQEEQTIIQLHALLGNRWSGIAAHLPKRT GhMML9_Dt(TM-1_HAU)
MGRSPCCDKVGLKKGPWTPEEDQKLLAYIEQHGHGSWRALPAKAGLQRCGKSCRLRWINYLRPDIKRGKFSLQEEQTIIQLHALLGNRWSGIAAHLPKRT GhMML9_Dt(TM-1_JGI)
MGRSPCCDKVGLKKGPWTPEEDQKLLAYIEQHGHGSWRALPAKAGLQRCGKSCRLRWINYLRPDIKRGKFSLQEEQTIIQLHALLGNRWSGIAAHLPKRT GrMML9(G. raimondii)
MGRSPCCDKVGLKKGPWTPEEDQKLLAYIEQHGHGSWRALPAKAGLQRCGKSCRLRWINYLRPDIKRGKFSLQEEQTIIQLHALLGNRWSGIAAHLPKRT GaMML9(G. arboreum)
                                        DNA-binding motif 1

        110       120       130       140       150       160       170       180       190       200
....|....|....|....|....|....|....|....|....|....|....|....|....|....|....|....|....|....|....|....|
DNEIKNYWNTHLKKRLNKMGIDPVTHKPKTNALGSATGNPKDAATLSHMAQWESARLEAEARLVRESKLVRFSSSSSS-SSSSAPQHTSNPVMTPPATRP GbMML9_Dt(Hai7124)
DNEIKNYWNTHLKKRLNKMGIDPVTHKPKTNALGSATGNPKDAATLSHMAQWESARLEAEARLVRESKLVRFSSSSSS-SSSSAPQHTSNPVMTPPATRP GbMML9_Dt(3-79)
DNEIKNYWNTHLKKRLNKMGIDPVTHKPKTNALGSATGNPKDAATLSHMAQWESARLEAEARLVRESKLVRFSSSSSS-SSSSAPQHTSNPVMTPPATRP GhMML9_Dt(TM-1_ZJU)
DNEIKNYWNTHLKKRLNKMGIDPVTHKPKTNALGSATGNPKDAATLSHMAQWESARLEAEARLVRESKLVRFSSSSSS-SSSSAPQHTSNPVMTPPATRP GhMML9_Dt(TM-1_HAU)
DNEIKNYWNTHLKKRLNKMGIDPVTHKPKTNALGSATGNPKDAATLSHMAQWESARLEAEARLVRESKLVRFSSSSSS-SSSSAPQHTSNPVMTPPATRP GhMML9_Dt(TM-1_JGI)
DNEIKNYWNTHLKKRLNKMGIDPVTHKPKTNALGSATGNPKDAATLSHMAQWESARLEAEARLVRESKLVRFSSSSSS-SSS-APQHTSNPVMTPPATRP GrMML9(G. raimondii)
DNEIKNYWNTHLKKRLNKMGIDPVTHKPKTNALGSATGNPKDAATLSHMAQWESARLEAEARLVRESKFVGSSSSSSSSSSSSAPQHTSNTVMTPPATRP GaMML9(G. arboreum)
DNA-binding motif 2
```

```
                210       220       230       240       250       260       270       280       290       300
       ....|....|....|....|....|....|....|....|....|....|....|....|....|....|....|....|....|....|....|....|
       QCLDVLKAWQGLVTGLFTFNNTTDNLQSPTSTLNFVENTNTLANGLINDNSMELHEMGAWFRQDSSYRAVENMNMEDYSDMMVWESGDHQQCSSMAAPAE  GbMML9_Dt(Hai7124)
       QCLDVLKAWQGLVTGLFTFNNTTDNLQSPTSTLNFVENTNTLANGLINDNSMELHEMGAWFRQDSSYRAVENMNMEDYSDMMVWESGDHQQCSSMAAPAE  GbMML9_Dt(3-79)
       QCLDVLKAWQGLVTGLFTFNNTTDNLQSPTSTLNFVENTNTLANGLINDNSMELHEMGAWFRQDSSYRAVENMNMEDYSDMMVWESGDHQQCSSMAAPAE  GhMML9_Dt(TM-1_ZJU)
       QCLDVLKAWQGLVTGLFTFNNTTDNLQSPTSTLNFVENTNTLANGLINDNSMELHEMGAWFRQDSSYRAVENMNMEDYSDMMVWESGDHQQCSSMAAPAE  GhMML9_Dt(TM-1_HAU)
       QCLDVLKAWQGLVTGLFTFNNTTDNLQSPTSTLNFVENTNTLANGLINDNSMELHEMGAWFRQDSSYRAVENMNMEDYSDMMVWESGDHQQCSSMAAPAE  GhMML9_Dt(TM-1_JGI)
       QCLDVLKAWQGLVTGLFTFNNTTDNLQSPTSTLNFVENTNTLANGLINDNSMELHEMGAWFRQDSSYRAVENMNMEDYSDMMVWESGDHQQCSSMAAPAE  GrMML9(G. raimondii)
       QCLDVLKAWQGLVTRLFTFNNTTDNLQSPTSTLNFVENTNTLANGLINENSMELHEMGAWFRQDSSYRAVENMDMEDYSDMMVWESGDHQQWSSMAAPAE  GaMML9(G. arboreum)

                310       320       330
       ....|....|....|....|....|....|....|
       NLNETSYG--N-SSSSSSSLEENRNYWNNILNLVS                                                                    GbMML9_Dt(Hai7124)
       NLNETSYG--N-SSSSSSSLEENRNYWNNILNLVS                                                                    GbMML9_Dt(3-79)
       NLNETSYG--SSSSSSSSSLEENRNYWNNILNLVS                                                                    GhMML9_Dt(TM-1_ZJU)
       NLNETSYG--SSSSSSSSSLEENRNYWNNILNLVS                                                                    GhMML9_Dt(TM-1_HAU)
       NLNETSYG--SSSSSSSSSLEENRNYWNNILNLVS                                                                    GhMML9_Dt(TM-1_JGI)
       NLNETSYGNSSSSSSSSSSLEETRNYWNNILNLVS                                                                    GrMML9(G. raimondii)
       NLNETSYG--N-SSSSSSSLEENRNYWNNILNLVS                                                                    GaMML9(G. arboreum)
```

Variations between Gh and Gb
