# Supplementary material for: Open-Bud Duplicate Loci Are Identified as MML10s, Orthologs of MIXTA-Like Genes on Homologous Chromosomes of Allotetraploid Cotton
Source: Front Plant Sci. 2020 Feb 18;11:81. doi: 10.3389/fpls.2020.00081 (PMC7040098; doi:10.3389/fpls.2020.00081)
Supplement: Supplementary file 1 [file DataSheet_1.zip › Figure S3.pdf]

Figure S3 Alignment of promoter sequences of *MML10* from Gh, Gb, *G. arboreum* and *G. raimondii*. The predicted transcription factors binding-sites are framed.

```
                  10        20        30        40        50        60        70        80        90       100
          ....|....|....|....|....|....|....|....|....|....|....|....|....|....|....|....|....|....|....|....|
          CATATCGTGCCGTTACGACACATA-----TCAGATAAT--ATGCAGCCAAGCTGCCAAAATATAGGCGATGA------TCGCCATACAGAA--------T GhMML10_At(TM-1)
          CATATCGTGCCGTTACGACACATA-----TCAGATAAT--ATGCAGCCAAGCTGCCAAAATATAGGCGATGA------TCGCCATACAGAA--------C GbMML10_At (3-79)
          CATATCGTGTCGTTACGACACATA-----TCAGATAAT--ATGCAGCCAAGCTGCCAGAATATAGGCGATGA------TCGCCATACAGAA--------C GaMML10(Shixiya1)
          CATATTGATTTCTCGAGATGAAGA-TTTTTCTAAAAATTAATGAAGGAAATATTCGAAGTTTGGGATTTTGAGAAATTGTGCCCTAATGTATTGGGCTGC GhMML10_Dt (TM-1)
          CATATTGATTTCTCGAGATGAAGATTTTTTCTAAAAATTAATGAAGGAAATATTCGAAGTTTGGGATTTTGAGAAATTGTGCCCTAATGTATTGGGCTGC GbMML10_Dt (3-79)
          CGTATTGATTTCTCGAGATGAATATTTTTTCCAAAAATTAATGAAGGAAATATTCGAAGTTTAGGATTTTGAGAAATTGTGCCCTAATGTATTGGGCTGC GrMML10

                 110       120       130       140       150       160       170       180       190       200
          ....|....|....|....|....|....|....|....|....|....|....|....|....|....|....|....|....|....|....|....|
          ACTTCCTCCACATATATAAATCCCACTCCAATCAGATATACATAATCAAAATT------ACCATGCTT----ACATGCTCATATTCAAATATAAAAAATA GhMML10_At(TM-1)
          ACTTCCTCCACATATATAAATCCCACTCCAATCAGATATACATAATCAAAATT------ACCATGCTT----ACATGCTCATATTCAAATATCAAAAATA GbMML10_At (3-79)
          ACTTCCTCCACATATATAAATCCCACTCCAATCAGATATACATAATCAAAATT------ACCATGCTT----ACATGCTCATATTCAAATATCAAAAATA GaMML10(Shixiya1)
          GGTTTCTTCATAAATTTTAA-------ACAATTGAATGTTCTT--TTAAATTTTATTACACAAGTCTTTTGGACAAACTCATTTTTAAAGGAATAAAATA GhMML10_Dt (TM-1)
          GGTTTCTTCATAAATTTTAA-------ACAATTGAATGTTCTT--TTAAATTTTATTACACAAGTCTTTTGGACAAACTCATTTTTAAAGGAATAAAATA GbMML10_Dt (3-79)
          GGTTTCTTCATAAATTTTAA-------ACAATTGAATGTTCTT--TTAAATTTTATTACACAAGTCTTTTGGACAAACTCATTTTTAAAGGAATAAAATA GrMML10

                 210       220       230       240       250       260       270       280       290       300
          ....|....|....|....|....|....|....|....|....|....|....|....|....|....|....|....|....|....|....|....|
          T-ATATATCAGCCTAATTAGACATG---------------CATAACAGTGTCTTACTCAGAGCAGAACGTCAGACCACCATATCACATAGTTTTAGGGTT GhMML10_At(TM-1)
          T-ATATATCAGCCTAATTAGACATG---------------CATAACAGTGTCTTACTCAGAGCAGAACGTCAGACCACCAGATCACATAGTTTTAGGGTT GbMML10_At (3-79)
          T-ATATATAAGCCTAATTAGACATA---------------CATAACAGTGTCTTACTCAGAGCAGAATGTCAAACCACCAGATCACATAGTTTTAGGGTT GaMML10(Shixiya1)
          TCGTGCACTAACGC-ATTGGGTGTGATATTTTTTCTCCGAAATAAGAGAGTCTTAATTA----ATAACTTGATTTAATTAAGGATCGTATTTTTAACTTT GhMML10_Dt (TM-1)
          TCGTGCACTAACGC-ATTGGGTGTGATATTTTTTCTCCGAAATAAGAGAGTCTTAATTA----ATAACTTGATTTAATTAATGATCGTATTTTTAACTTT GbMML10_Dt (3-79)
          TCGTGCACTAACGC-ATTGGGTGTGACATTTTTTCTCCAAAATGAGAGAGTCTTAATCA----ATAACTTGATTTAATTAAGGATCGTATTTTTAACTTT GrMML10

                 310       320       330       340       350       360       370       380       390       400
          ....|....|....|....|....|....|....|....|....|....|....|....|....|....|....|....|....|....|....|....|
          TGG---TTAGCCTTTACTGACCATACAATAGGCCCAAAGTCAATCGGAACGACCCGTGCAACCCTAGGAAAAATTTTAAAATTATGGGCCCACATGCCCG GhMML10_At(TM-1)
```

```
TGG---TTAGCCTTTACTGACCATACAATAGGCCCAAAGTCAATCGGAACGACCCGTGCAACCCTAGGAAAAATTTTAAAATTATGGGCCCACATGCCCG GbMML10_At (3-79)
TGG---TTAGCCTTTACTGACCATACGATAGGCCCAAAATCAATCGGAATAACCCGTGCAACCCTAGGAAAAATTTTAAAATTATGGGCCCACATGACCG GaMML10(Shixiya1)
CGACATTTAGACATT----------------------AATTAATC---------------AACTTGGTACCAATTTT-------TGGG------------ GhMML10_Dt (TM-1)
CGGCATTTAGACATT----------------------AATTAATC---------------AACTTGGTACCAATTTT-------TGGG------------ GbMML10_Dt (3-79)
TGGCATTTAGACATT----------------------AATTAATC---------------AACTTGGTACCAATTTT-------TGGG------------ GrMML10

        410       420       430       440       450       460       470       480       490       500
....|....|....|....|....|....|....|....|....|....|....|....|....|....|....|....|....|....|....|....|
TACGAGCTCACACGCTCGTGTGGCCCACATGACCTGGCTCAATACTCACACACCCGTGTGGCCTACACAACCTGGCCTAATACCCATACACCCAACTTGG GhMML10_At(TM-1)
TACGAGCTCACACGCTCGTGTGGCCCACATGACCTGGCCCAATACTC----------------------------------------------------- GbMML10_At (3-79)
TACGAGCTCACACGCTCGTGTGGCCCACATGACCTGGCCCAATACTCACACACCCGTGTGGCCTACACAACCTGGCCCAATACCCATACACCCAACTTGG GaMML10(Shixiya1)
------------CGTTAGGGAAGTGCTAAT---------------------------------------------------------------------- GhMML10_Dt (TM-1)
------------CGTTAGGGAAGTGCTAAT---------------------------------------------------------------------- GbMML10_Dt (3-79)
------------CGTTAGGGAAGTGCTAAT---------------------------------------------------------------------- GrMML10

        510       520       530       540       550       560       570       580       590       600
....|....|....|....|....|....|....|....|....|....|....|....|....|....|....|....|....|....|....|....|
CCTAGCCCGTGTGGCCCACACGACCACACCCACATTATCACACGACCGTGTCTTACGCACGACCATGCCCTCATCAAACACACGGTCGTGTCTCGCACAC GhMML10_At(TM-1)
CCTAGCCCGTGTGGCCCACACGACCACACCCACATTATCACACGACCGTGTCTTACGCACGACCATGCCCTCATCAAACACACGGTCGTGTCTCGCACAC GbMML10_At (3-79)
CCTAGCCCGTGTGGCCCACACGACCACACCCATATTATCACACGACCGTGTCTTACGCACGACCATGCCCTCATCAAACACACGGTCGTGTCTCGCACAC GaMML10(Shixiya1)
CCTTCCTCGTACGTAACTGACTCCCGAACCTGTTTTTTCTAAGACTCGTA----------GAC-----------------CAAAGTCGTTTTTAG----- GhMML10_Dt (TM-1)
CCTTCCTCGTACGTAACTGACTCCCGAACCCGTTTTTTCTAAGACTCGTA----------GAC-----------------CAAAGTCGTTTTTAG----- GbMML10_Dt (3-79)
CCTTCCTCGTACGTAACTGACTCCCAAACCCGTTTTTTCTAAGACTCGTA----------GAC-----------------CAAAGTCGTTTTTAG----- GrMML10

        610       620       630       640       650       660       670       680       690       700
....|....|....|....|....|....|....|....|....|....|....|....|....|....|....|....|....|....|....|....|
GGCCACCCAAACAGGCGGCCACACTCCCATGTGGCATCGAAAGTAGACATTTTTGGCTTTCGCTGAAACCTCATTTTCCATAAATTGGGAAC--ACACCT GhMML10_At(TM-1)
GGCCACCCAAACAGGCGGCCACACTCCCGTGTGGCATCGAAAGTAGACATTTTTGGCTTTCGCTGAAACCTCATTTTCCATAAATTGGGAAC--ACACCT GbMML10_At (3-79)
-GCCACCCAAACAGGTGGCCACACTCCCGTGTGGCATCGAAAGTAGACATTTTTGGCTTTCGCTGAAACTTCATTTTCCATAAATTGGGAAC--ACACCT GaMML10(Shixiya1)
-GTGATCCAATCAGACCTCAATA---------------------AAAGATTGGTGATGACTCCCAATTTTTCGTTTT---TAAAT----AATTAATTTTT GhMML10_Dt (TM-1)
-GTGATCCAATCAGACCTCAATA---------------------AAAGATTGGTGATGACTCCCAATTTTTCGTTTT---TAAATCGATAATTAATTTTT GbMML10_Dt (3-79)
-GTGATCCAATCAGACATCAATA---------------------AAAGATTGGTGGCGACTCCCAATTTTTCGTTTT---TAAATCGATAATTAATTTTT GrMML10

        710       720       730       740       750       760       770       780       790       800
```

```
....|....|....|....|....|....|....|....|....|....|....|....|....|....|....|....|....|....|....|....|
GTTTCAATTTTGATGCGAGCACACACCTGAGATCACCAGAATCTAAAACCGACAAACCAAACTCCAAAATCATTC--ATAATTCATGATTAA--ACCAAA GhMML10_At(TM-1)
GTTTCAATTTTGATGCGAGCACACACCTGAGATCACCAGAATCTAAAACCGACAAACCAAACTCCAAAATCATTC--ATAATTCATGATTAA--ACCAAA GbMML10_At (3-79)
GTTTCAATTTTGATACGAGCACACACCTGAGATCACCAGAATCTAAAACCGACAAACCAAACTCCAAAATCATTC--ATAATTCATGATTAA--ACCAAA GaMML10(Shixiya1)
GTTTTGTTTCT---------------------TCAAAAAAAAATGGTTTCGACAAAATTTA-TTAAATATTATCCTAAAAAATACTGATGAATTATAAAA GhMML10_Dt (TM-1)
GTTTTGTTTCT---------------------TCAAAAAAAAATGGTTTCGACAAAGTTTA-TTAAATATTATCCTAAAAAATACTGATGAATTATAAAA GbMML10_Dt (3-79)
GTTTTGTTTCT---------------------TCAAAAAAA--TGGTTTCGACAAAGTTTA-TTAAATATTATCCTAAAAAATACTAATGAATTATAAAA GrMML10

       810       820       830       840       850       860       870       880       890       900
....|....|....|....|....|....|....|....|....|....|....|....|....|....|....|....|....|....|....|....|
ACTACCAAAATCAAAAATAGTCCAATTCAAACGTTCATCACTTACCCCAACACTTCGAA-----CGATTTCAACTACGATTTCGCAAGA-----GAAGAG GhMML10_At(TM-1)
ACTACCAAAATCAAAAATAGTCCAATTCAAACGTTCATCACTTACCCCAACACTCCGAA-----CGATTTCAACTACGATTTCGCAAGA-----GAAGAG GbMML10_At (3-79)
ACTACCAAAATCAAAAATAGTTCAATTCAAACGTTCATCACTTACCCCAACACACCGAA-----CGATTTCAACTATGATTTCGCAAGA-----GAAGAG GaMML10(Shixiya1)
AGTTCTAAAAT---ATATAG-------AAAAATTTTGTTATTT----TGATGGTTTGAAGTATTTGATTTCGGTAATAATG-AATGAGGAACTTGAAGTG GhMML10_Dt (TM-1)
AGTTCTAAAAT---ATATAG-------AAAAATTTTGTTATTT----TGATGGTTTGAAGTATTTGATTTCGGTAATAATG-AATGAGGAACTTGAAGTG GbMML10_Dt (3-79)
AGTTCTAAAAT---ATATAG-------AAAATTTTTGTTATTT----TGATGGTTTGAAGTATTTGATTTCGGTAATAATGAAATGAGGAGCTTGAAGTG GrMML10

       910       920       930       940       950       960       970       980       990      1000
....|....|....|....|....|....|....|....|....|....|....|....|....|....|....|....|....|....|....|....|
CCCCATTCTTCGCGATTCG---TTGCTGTCAAA------ACACGAGT----------TCAACATCAACAAAAAGTTCAACACTCTATAAACGAATT---- GhMML10_At(TM-1)
CCCCATTCTTGGCGATTCG---TTGCTGTCAAA------ACACGAGT----------TCAACATCAACGAAAAGTTCAACACTCTATAAACGAATT---- GbMML10_At (3-79)
CCCCATTCTTCGCGATTCG---TTGCTGTCAAA------ACACGAGT----------TCAACATCAACGAAAAGTTCAACACTCTATAAACGAATT---- GaMML10(Shixiya1)
CGATAGTGATTG-GACTTGTATTGGGTGTCGAATGAGTGATTTGAGTGTTTTGTGGCTCAAGGTGAATAAAGAGT-------------AATGAATTTGGT GhMML10_Dt (TM-1)
CGATAGTGATTG-GACTTGTATTGGGTGTCGAATGAGTGATTTGAGTGTTTTGTGGCTCAAGGTGAATAAAGAGT-------------AATGAATTTGGT GbMML10_Dt (3-79)
CGATAGTGATTG-GACTTGTATTGGGTGTCGAATGAGTGATTTGAGTGTTTTGTGGCTCAAGGTGAATAAAGAGT-------------AATGAATTTGGT GrMML10

      1010      1020      1030      1040      1050      1060      1070      1080      1090      1100
....|....|....|....|....|....|....|....|....|....|....|....|....|....|....|....|....|....|....|....|
---AAAGAAAGACTCCCTACTCGAGCAAAACATACTACCCTTACTTACCAAACGTATAACCACAATGATACGAAACATCGCAATAT-TGAAGAA------ GhMML10_At(TM-1)
---AAAGAAAGACTCCCTACTCGAGCAAAACATACTACCCTTACTTACCAAACGTATAACCACAATGATACGAAACACCGCAATAT-TGAAGAA------ GbMML10_At (3-79)
---AAAGAAAGACTCTCTACTCGAGCAAAACATACTACCCTTACTTACCAAACGTATAACCACAATGATACGAAACACCGCAATAT-TGAAGAA------ GaMML10(Shixiya1)
TGAGAAGAAATGAT----------ACAAGAGAAGCT------------CATAAGCATGA--ACTAAGGTATGATATAGGGCAACATGTGAAGGAAAACAA GhMML10_Dt (TM-1)
TGAGAAGAAATGAT----------ACAAGAGAAGCT------------CATAAGCATGA--ACTAAGGTATGATATAGGGCAACATGTGAAGGAAAACAA GbMML10_Dt (3-79)
TGAGAAGAAATGAT----------ACAAGAGAAGCT------------CATAAGCATGA--ACTAAGGTATGATATAGGGCAACATGTGAAGGAAAACAA GrMML10
```

```
              1110      1120      1130      1140      1150      1160      1170      1180      1190      1200
         ....|....|....|....|....|....|....|....|....|....|....|....|....|....|....|....|....|....|....|....|
--------CAATTTTTATTAAA--ACGAAAAAGAAAAATGAGAAAACTTCTGTTGAAAAGAAAAGGGGAAAAATAACAGAGGAGGAAAAAAAGGAAACGT GhMML10_At(TM-1)
--------CAATTTTTATTAAA--ACGAAAAAGAAAAATGAGAAAACTTCTGTTGAAAAGAAAAGGGGAAAAATAACAGAGGAGGAAAAAAAGGAAACGT GbMML10_At (3-79)
--------CAATTTTTATTAAA--ACGAAAAAGAAAAATGAGAAAACTTCTGTTGAAAAGAAAAGGGGAAAAATAACAGAGGAGGAAAAAAAGGAAACGT GaMML10(Shixiya1)
GAATTAGTAAATTTTTATTAAAATACTGCAAAAAAAAT-----AGAATATTATCAAAAAGAAAATACAAAAAAATATATAAATTATTAAAAATAATATTA GhMML10_Dt (TM-1)
GAATTAGTAAATTTTTATTAAAATACTGCAAAAAAAAAT----AGAATATTATCAAAAAGAAAATACAAAAAAATATATAAATTATTAAAAATAATATTA GbMML10_Dt (3-79)
GAATTAGTAAATTTTTATTAAAATACTGCAAAAAAAT------AGAATATTATCAAAAAGAAAATACAAAAA---ATATAAATTATTAAAAATAATATTA GrMML10

              1210      1220      1230      1240      1250      1260      1270      1280      1290      1300
         ....|....|....|....|....|....|....|....|....|....|....|....|....|....|....|....|....|....|....|....|
CAG-TTTTT-----TTCCAAGAAGGGAGAGATTTTTGGGA--AAACAAATTTTACCCAAA-TCTCACTACATCCACATCC-----ATAATCACCCAATAA GhMML10_At(TM-1)
CAG-TTTTT-----TTCCAAGAAGGGAGAGATTTTTGGGA--AAACAAAATTTACCCAAA-TCTCACTACATCCACATCC-----ATAATCACCCAATAA GbMML10_At (3-79)
CAG-TTTTT-----TTCCAAGAAGGGAGAGATTTTGGGAA---AACAAATTTTACCCAAA-TCTCACTACATCCACATCC-----ATAATCACCCAATAA GaMML10(Shixiya1)
CTA-CTTTTATAAATTTTATAATTTTAAATATTTTT---------TATATTTTTTATAAATTTTTAATATTTTTTTATCTTTTTAGTAATCTTCATATAA GhMML10_Dt (TM-1)
CTA-CTTTTATAAATTTTATAATTTTAAATATTTTAAATATTTTTTATATTTTTTATAAATTTTTAATATTTTTTTATCTTTTTAGTAATCTTCATATAA GbMML10_Dt (3-79)
CTACTTTTTATAAATTTTATAATTTTAA------------------ATATTTTTTATAAATTTTTAATATTTTTTTATCTTTTTAGTAATCTTCATATAA GrMML10

              1310      1320      1330      1340      1350      1360      1370      1380      1390      1400
         ....|....|....|....|....|....|....|....|....|....|....|....|....|....|....|....|....|....|....|....|
CTCAGAATCCCACTACCACTGAACTTCTAACCACCACCGAAGCAAAAAAATATATATTAA--CGACATTCACGCAAGGACTCAAA-----------CACA GhMML10_At(TM-1)
CTCAGAATCCCACTACCACTGAACTTCTAACCACCACCGAAGCAAA--AAAAAATATTAA--CGACATTCACGCAAGGACTCAAA-----------CACA GbMML10_At (3-79)
CTCAGAATCCCACTACCACTGAACTTCTAACCATCACCGAAGCAAAAAAAAAAAAATATTAACGACATTCACACAATGACTCAAAA----------CACA GaMML10(Shixiya1)
TTTTAAATATC----CAATTGGTTGATTAA--------AAAGGAAAATAGAAAAGGTTAAACTAATATTAATAAATAAATTTTAAACCAACAGTGTCGTA GhMML10_Dt (TM-1)
TTTTAAATATC----CAATTGGTTGATTAA--------AAAGGAAAATAGAAAAGGTTAAACTAATATTAATAAATAAATTTTAAACCAACAGTGTCGTA GbMML10_Dt (3-79)
TTTTAAATATC----CAATTGGTTGATTAA--------AAAGGAAAATAGAAAAAGTTAAACTAATATTAATAAATAAATTTTAAACCAACAGTGTCGTA GrMML10

              1410      1420      1430      1440      1450      1460      1470      1480      1490      1500
         ....|....|....|....|....|....|....|....|....|....|....|....|....|....|....|....|....|....|....|....|
AAATTTTCAACACACTAACT-CCTT---------ACCACTCGAATCAATAGGCTCATTCTGATATGGACTAACA-----------GACAATTTAATATAA GhMML10_At(TM-1)
AAATTTTCAACACACTAACT-CCTT---------ACCACTCGAATCAATAGGCTCATTCTGATATAGACTAACA-----------GACAATTTAATATAA GbMML10_At (3-79)
AAATTT-CAACACACTAACT-CCTT---------ACCACTCAAATCAATAGGCTCATTCTGATATGGACTAACA-----------GACAATTTAATATAA GaMML10(Shixiya1)
AATTTTATAACGAGTTAA---------------------------------------------------------------------------------- GhMML10_Dt (TM-1)
```

```
AATTTTTTAACGAGTTAAAT-AATTTTTGGAAGGATCAATTATGAGAATAATATTATAACTATATATTTAAAAATTTCGAGAGGCGAAACATAAGTATAG GbMML10_Dt (3-79)
AATTTTATAACGAGTTAAATAATTTTTTGGAAGGATCAATTATGAGAATAATATTATAACTATATATTTAAAAATTTCGAGAGGCGAAACATAAGTATAG GrMML10

                1510      1520      1530      1540      1550      1560      1570      1580      1590      1600
....|....|....|....|....|....|....|....|....|....|....|....|....|....|....|....|....|....|....|....|
GCTCACTCAACAAGGGTAAGGCT--TGAATTAGAAAAATAACAAAATTT--------------------------------------------------- GhMML10_At(TM-1)
GCTCACTCAACAAGGGTAAGGCT--TGAATTAGAAAAATAACAAAATTT--------------------------------------------------- GbMML10_At (3-79)
GCTCACTCAACAAGGGTAAGGCT--TGAATTAGAAAAATAATAAAATTT--------------------------------------------------- GaMML10(Shixiya1)
-------TAATAATAATAA--------------------------------------------------------------------------------- GhMML10_Dt (TM-1)
TTTCTTTTAA-AACGGTAATATGAATAATATAGAAACACTTTATA[ATTTTGGGGGGGGGGGGGGGGGGGTGGGGGA]TGGCTAATAGAGACGCACACAAAG GbMML10_Dt (3-79)
TTTCTTTTAA-AACGGTAATATGAATAATATAGAAACACTTTATA[ATTTTGGGGGGGGGAGGGGGG----GTGGGG]TGGCTAATAGAGACGCACACAAAG GrMML10
                                                [MYB, C2H2, TALE, ERF, BBR-BPC]

                1610      1620      1630      1640      1650      1660      1670      1680      1690      1700
....|....|....|....|....|....|....|....|....|....|....|....|....|....|....|....|....|....|....|....|
----GACAAATGTGAGGCTTGAACCCAAGACCTCACACACACTCCTAAAATA---------------TTTAATCA------------------------- GhMML10_At(TM-1)
----GACAAATGTGAGGCTTGAACCCAAGACCTCACACACACTCCTAAAATA---------------TTTAATCA------------------------- GbMML10_At (3-79)
----GACAAATGTGAGGCTTGAACCCAAGACCTCACACACACTCCTAAAATA---------------TTTAATCA------------------------- GaMML10(Shixiya1)
---------------------------------------------------------------------------------------------------- GhMML10_Dt (TM-1)
GAGAGACTAATGGCAGACATGAAATTGGAAATAAATATTTGCTCTAAGGATAAAAGTATCGTGAAGCCTTTATTAGGAGTTAAATTGTACTTTGTTTTTT GbMML10_Dt (3-79)
GAGAGACTAATGGCAGACATGAAATTGGAAATAAATATTTGCTCTAAGGATAAAAGTATCGTGAAGTTTTTATTAGGAGTTAAATTGTACTTTG-TTTTT GrMML10

                1710      1720      1730      1740      1750      1760      1770      1780      1790      1800
....|....|....|....|....|....|....|....|....|....|....|....|....|....|....|....|....|....|....|....|
---CTAAAATAAATACA--------------------------------------CATTTGTGTTAAATATTTA----------------------CAGA GhMML10_At(TM-1)
---CTGAAATAAATACA--------------------------------------CATTTGTGTTAAATATTTA----------------------CAGA GbMML10_At (3-79)
---CTGAAATAAATACA--------------------------------------CATTTGTGTTAGATATTTA----------------------CAGA GaMML10(Shixiya1)
---------------------------------------------------------------------------------------------------- GhMML10_Dt (TM-1)
TATTTAAAATAGGTAAATTAATTATGGAACGTTAGATGAAAGAGAAAATAAATTTTTTTTTTGTTAAAAATTCAATTTATTTCTACTGTTAAAAATTAGT GbMML10_Dt (3-79)
TATTTAAAATAGGTAAATTAATCATGGAACGTTAGATGAAAGAGAAAATAAG--TTTTTTTTATTAAAAATTCAATCTATTTCTACTGTTAAAAATTAGT GrMML10

                1810      1820      1830      1840      1850      1860      1870      1880      1890      1900
....|....|....|....|....|....|....|....|....|....|....|....|....|....|....|....|....|....|....|....|
ATTAGAAATAAATTATTCAAAGAGTTACA----------------------------------------------------------------------- GhMML10_At(TM-1)
```

```
ATTAGAAATAAATTATTCAAAGAGTTACA----------------------------------------------------------------------- GbMML10_At (3-79)
ATTAGAAATAAATTATTCAGAGCGTTACA----------------------------------------------------------------------- GaMML10(Shixiya1)
---------------------------------------------------------------------------------------------------- GhMML10_Dt (TM-1)
ATGGGTGACAGAATAATCAAACAGTTACACTTAGGGTGCTACATGTATCTCATTCTAATGTACAAAGACCAGTTTTTAACAATAGAAATGCATAGATTTT GbMML10_Dt (3-79)
ATGGGTGACAGAATAATCAAACAGTTACACCTAGGGTGCCACGTGTATCTCATTCTAATGTATAAAGGCCAGTTTTTAACAATAGAAATGCATAGATTTT GrMML10

        1910      1920      1930      1940      1950      1960      1970      1980      1990      2000
....|....|....|....|....|....|....|....|....|....|....|....|....|....|....|....|....|....|....|....|
------------------------------------------------------------------------------------------------AAGG GhMML10_At(TM-1)
------------------------------------------------------------------------------------------------AAGG GbMML10_At (3-79)
------------------------------------------------------------------------------------------------AAGG GaMML10(Shixiya1)
---------------------------------------------------------------------------------------------------- GhMML10_Dt (TM-1)
TAATAGAAGAATCAATTTGTTCTTGAATCTAACGTATAGAGACTAATTTAAA-TTTTTTTTAGTATAGGGGTAAAATGTAATCTAATCCCTAATATAATG GbMML10_Dt (3-79)
TAATAGAAGAACCTATTTGCTCTTGAATCTAACGTATAGAGACTAATTTAAATTTTTTTTTAATATAGGGATAAAATGCAATCTAATCCCTAATATAAGG GrMML10

        2010      2020      2030      2040      2050      2060      2070      2080      2090      2100
....|....|....|....|....|....|....|....|....|....|....|....|....|....|....|....|....|....|....|....|
GTATAAAGACCTCTATAGTACTTTTACCTTTACTAACAACATTAAAGTAATTTTAAATAGAG------------------------ATAATAATAATAAT GhMML10_At(TM-1)
GTATAAAGACCTCTATAGTACTTTTACCTTTACTAACAACATTAAAGTAATTTTAAATAGAG------------------------ATAATAATAATAAT GbMML10_At (3-79)
GTATAAAGACCTCTATAGTACTTTTACCTTTACTAACAACATTAAAGTAATTTTAAATAGAG------------------ATAATAATAATAATAATAAT GaMML10(Shixiya1)
------------------------------TAATAATAATAATAA---------------------------------TAATAATAATAATAATAATAAT GhMML10_Dt (TM-1)
GTATAAGGACCTCTATGATACTTTTACCTTTACTGGCAACATTAAAGTAATTTTAAATAGAA---------ATGATAATAATAATAATAATAATAATAAT GbMML10_Dt (3-79)
GTATAAGGACCTCTATGATACTTTTACCTTTAC-GGCAACATTAAAGTAATTTTAAATAGAGATAATAATAATAATAATAATAATAATAATAATAATAAT GrMML10

        2110      2120      2130      2140      2150      2160      2170      2180      2190      2200
....|....|....|....|....|....|....|....|....|....|....|....|....|....|....|....|....|....|....|....|
AATAATGTTTAAATCTTGGAGTTCTAAACAATTCCTGTCCTCTATCTCTAACAAAATAATGAGCTTGAGTTTAGTTTTAATTTCATTTTATATATTTATC GhMML10_At(TM-1)
AATAATGTTTAAATCTTGGAGTTCTAAACAATTCCTATCCTCTATCTCTAACAAAATAATGAGCTTGAGTTTAGTTTTAATTTCATTTTATATATTTATC GbMML10_At (3-79)
AATAATGTTTAAATCTTGGAGTTCTAAACAATTCCTATCCTCTATCTCTAACAAAATTATGAGCTTGAGTTTAGTTTTAATTTCATTTTATATATTTATC GaMML10(Shixiya1)
AATAGTGTTTAAATCTTGGATTTCTAAACAATTCCTATCCTCTATCTCTAACAAAATTATGAGCTAGAGTTTATTTGTAATTTGATTTTATATATTTATC GhMML10_Dt (TM-1)
AATAGTGTTTAAATCTTGGATTTCTAAACAATTCCTATCCTCTATCTCTAACAAAATTATGAGCTAGAGTTTATTTTTAATTTGATTTTATATATTTATC GbMML10_Dt (3-79)
AATAATGTTTAAATCTTGGATTTCTAAACAATTCCTATCCTCTATCTCTAACAAAATTATGAGCTAGAGTTTAGTTTTAATTTGATTTTATATATTTATC GrMML10
                                                                     SNP between GhMML10_Dt and GbMML10_Dt
```

```
        2210      2220      2230      2240      2250      2260      2270      2280      2290      2300
....|....|....|....|....|....|....|....|....|....|....|....|....|....|....|....|....|....|....|....|
TAGATTAAAAAAAGATTACTTAAATAATCAACTTTTTCGTT--TTTTTTTTTTACGAAGTCTGAATAGTTGACAATGATTTACATCGCTATATTATTTTC GhMML10_At(TM-1)
TAGATTAAAAAAAGATTACTTAAATAATCAACTTTTTAGTTGTTTTTTTTTTTACGAAGTCTGAATAGTTGACAATGATTTACATCGCTATATTATTTTC GbMML10_At (3-79)
TAGATTAAAAAAAGATTACTTAAATAATCAACTTTTTAGT---TTTTTTTTTTACGAAGTCTGAATAGTTGACAATGATTTACATCGCTATATTA-TTTC GaMML10(Shixiya1)
TAGATTAAAAAAAGATTACTTAAATAATCAACTTTTTATTTTGTTTTTCTTTAACAAAGTCTGAATAGTTGACAATGATTTACATTGCTATATTATTTTC GhMML10_Dt (TM-1)
TAGATTAAAAAAAGATTACTTAAATAATCAACTTTTTATTTTGTTTTTCTTTAACAAAGTCTGAATAGTTGACAATGATTTACATTGCTATATTATTTTC GbMML10_Dt (3-79)
TAGATTAAAAAAAGATTACTTAAATAATCAACTTTTTATTTTGTTTTTCTTTAACAAAGTCTGAATAGTTGACAATGATTTACATTGCTATATTGTTTTC GrMML10

        2310      2320      2330      2340      2350      2360      2370      2380      2390      2400
....|....|....|....|....|....|....|....|....|....|....|....|....|....|....|....|....|....|....|....|
GCTGTGAATTAAGATATCAAAGAATAAAATGTTGAATATTTGGTATCTTACCCTA----TGTTATACACCAGTTAAGGAGATAATTGATTATTCAATTTA GhMML10_At(TM-1)
GCTGTGAATTAAGATATCAAAGAATAAAATGTTGAATATTTGGTATCTTACCCTA----TGTTATACACCAGTTAAGGAGATAATTGATTATTCAATTTA GbMML10_At (3-79)
GCTGTGAATTAAGATATCAAAGAATAAAATGTTGAATATTTGGTATCTTACCCTA----TGTTATACACCAGTTAAGGAGATAGTTGATTATTCAATTTA GaMML10(Shixiya1)
ACTGTGAATTAAGATATCAAAGAATAAAATGTTGAATAGTTGGTATCTTACCCTATGATTGTTATATACCAGTTAAGGAGACAATTGATTATTGAATTAA GhMML10_Dt (TM-1)
ACTGTGAATTAAGATATCAAAGAATAAAATGTTGAATAGTTGGTATCTTACCCTATGATTGTTATATACCAGTTAAGGAGACAATTGATTATTGAATTAA GbMML10_Dt (3-79)
ACTGTGAATTAAGATATCAAAGAATAAAATGTTGAATAGCTGGTATCTTACCCTATGATTGTTATATACCGGTTAAGGAGACAATTGATTATTGAATTAA GrMML10

        2410      2420      2430      2440      2450      2460      2470      2480      2490      2500
....|....|....|....|....|....|....|....|....|....|....|....|....|....|....|....|....|....|....|....|
GGACAGTCCTCTCGAGCCTTTTGATCTATCGTTGTCAATGTCATTAATGTTGGGATGAAGTGTAAAATTTATACTGGACACAGATAAAAGCATTCAGGTT GhMML10_At(TM-1)
GGACAGTCCTCTCGAGCCTTTTGATCTATCGTTGTCAATGTCATTAATGTTGGGATGAAGTGTAAAATTTATACTGGACACAGATAAAAGCATTCAGGTT GbMML10_At (3-79)
GGACAGTCCTCTCGAGCCTTTTGATCTATCGTTGTCAATGTCATTAATGTTGGGATGAAGTGTAAAATTTATACTGGACACAGATAAAAGCATTCAGGTT GaMML10(Shixiya1)
GGACAGCCCTCTCGAGCCTTTTGATCTATCGTTGTCAATGTCATTAATGTTGGAATGAAGTGTAAAATTTATACTGGACACAGATAAAAGCATTCGGGTT GhMML10_Dt (TM-1)
GGACAGCCCTCTCGAGCCTTTTGATCTATCGTTGTCAATGTCATTAATGTTGGAATGAAGTGTAAAATTTATACTGGACACAGATAAAAGCATTCGGGTT GbMML10_Dt (3-79)
GGACAGCCCTCTCGAGCCTTTTGATCTATCGTTGTCAATGTCATTAATGTTGGAATGAAGTGTAAAATTTATACTGGACACAGATAAAAGCATTCGGGTT GrMML10

        2510      2520      2530      2540      2550      2560      2570      2580      2590      2600
....|....|....|....|....|....|....|....|....|....|....|....|....|....|....|....|....|....|....|....|
TTAAACATGTTACAAGTGATTTTGAGTTTAGGTATTTGGATCCAATTATTTTAAATTTGAGTTCAAGTAAACTTAACTTCATTATATTTTTTACTTCTGG GhMML10_At(TM-1)
TTAAACATGTTACAAGTGATTTTGAGTTTAGGTATTTGGATCCAATTATTTTAAATTTGAGTTCAAGTAAACTTAACTTCATTATATTTTTTACTTCTGG GbMML10_At (3-79)
TTAAACATGTTACAAGTGATTTTGAGTTTAGGTATTTGGATCCAATTATTTTAAATTTGAGTTCAAGTAAACTTAACTTCATTATATTTTTTACTTCTGG GaMML10(Shixiya1)
TTAAACATGTTACAAGTGATTTTGAGTTTAGGTATTTGGGTCCAATTATTTTAAATTTGAGTTCAAGTAAACTTAACTTCATTATATTTTTTACTT-TGG GhMML10_Dt (TM-1)
TTAAACATGTTACAAGTGATTTTGAGTTTAGGTATTTGGGTCCAATTATTTTAAATTTGAGTTCAAGTAAACTTAACTTCATTATATTTTTTACTT-TGG GbMML10_Dt (3-79)
```

```
TTAAACATGTTACAAGTGATTTTGAGTTTAGGTATTTGGATCCAATTATTTTAAATTTGAGTTCAAGTAAACTTAACTTCATTATATTTTTTACTTCTGG GrMML10

               2610      2620      2630      2640      2650      2660      2670      2680      2690      2700
       ....|....|....|....|....|....|....|....|....|....|....|....|....|....|....|....|....|....|....|....|
CGGCTTTGAAATTTAACTAAATTCTAATTTTTAGATGTCAAAGTAGATTTTTCTTTCTATCATTTTTGAATTAAACAATTATTTTAAAGAAAACTTTGAA GhMML10_At(TM-1)
CGGCTTTGAAATTTAACTAAATTCTAATTTTTAGATGTCAAAGTAGATTTTTCTTTCTATCATTTTTGAATTAAACAATTATTTTAAAGAAAACTTTGAA GbMML10_At (3-79)
CGGCTTTGAAATTTAACTAAATTCTAATTTTTAGATGTCAAAGTAGATTTTTCTTTCTATCATTTTTGAATTAAACAATTATTTTAAAGAAAACTTTGAA GaMML10(Shixiya1)
CGGCTTTGAAATTTAACTAAATTCTAATTTTTAGATGTCAAAGTAGATTTTTCTTTCTATCATTTTTGAATTAAACAATTATTTTAAAGAAAACTTTGAA GhMML10_Dt (TM-1)
CGGCTTTGAAATTTAACTAAATTCTAATTTTTAGATGTCAAAGTAGATTTTTCTTTCTATCATTTTTGAATTAAACAATTATTTTAAAGAAAACTTTGAA GbMML10_Dt (3-79)
CGGCTTTGAAATTTAACTAAATTCTAATTTTTAGATGTCAAAGTAGATTTTTCTTTCTATCATTTTTGAATTAAACAATTATTTTAAAGAAAACTTTGAA GrMML10
  ERF

               2710      2720      2730      2740      2750      2760      2770      2780      2790      2800
       ....|....|....|....|....|....|....|....|....|....|....|....|....|....|....|....|....|....|....|....|
ATTAAATAAAAAT------AAATAAACAACCGGGGCCAACCCTTCTACCTGTCCCTTGGTTACACGTCTCTAAGCTAGCTGTATGCTCCCTTGGAATCTA GhMML10_At(TM-1)
ATTAAATAAAAAT------AAATAAACAACCGGGGCCAACCCTTCTACCTGTCCCTTGGTTACACGTCTCTAAGCTAGCTGTATGCTCCCTTGGAATCTA GbMML10_At (3-79)
ATTAAATAAAAAT------AAATAAACAACCGGGGCCAACCCTTCTACCTGTCCCTTGGTTACACGTCTCTAAGCTAGCTGTATGCTCCCTTGGAATCTA GaMML10(Shixiya1)
ATTAAATAAAAATTAAAAAAAATAAACAACCGGGGCCAACCCTTCTACCTGTCCCTTGGTTACACGTCTCTAAGCTAGCTGTATGCTCCCTTGGAATCTA GhMML10_Dt (TM-1)
ATTAAATAAAAATTAAAAAAAATAAACAACCGGGGCCAACCCTTCTACCTGTCCCTTGGTTACACGTCTCTAAGCTAGCTGTATGCTCCCTTGGAATCTA GbMML10_Dt (3-79)
ATTAAATAAAAATTAAAAAAAATAAACAACCAGGGCCAACCCTTCTACCTGTCCCTTGGTTACACGTCTCTAAGCTAGCTGTATGCTCCCTTGGAATCTA GrMML10
                                         TCP

               2810      2820      2830      2840      2850      2860      2870      2880      2890
       ....|....|....|....|....|....|....|....|....|....|....|....|....|....|....|....|....|....|....|...
AATTCTATAAATTGGTGAATCAATTTCCACCACTTAACAATCACAGATTCTTCTTCTATATTCCCTTTGGCAAAATACAAATATTAATATTAGGAATG GhMML10_At(TM-1)
AATTCTATAAATTGGTGAATCAATTTCCACCACTTAACAATCACAGATTCTTCTTCTATATTCCCTTTGGCAAAATACAAATATTAATATTAGGAATG GbMML10_At (3-79)
AATTCTATAAATTGGAGAATCAATTTCCACCACTTAACAATCACAGATTCTTCTTCTATATTCCCTTTGGCAAAATACAAATATTAATATTAGGAATG GaMML10(Shixiya1)
AATTCTATAAATTGGTGAATCAATTTCCACCACTTAACAATCGCAGATTCTTATTCTCTATTCTCTTTGGCAAAATACAAATATTAATATTAGGAATG GhMML10_Dt (TM-1)
AATTCTATAAATTGGTGAATCAATTTCCACCACTTAACAATCGCAGATTCTTATTCTCTATTCTCTTTGGCAAAATACAAATATTAATATTAGGAATG GbMML10_Dt (3-79)
AATTCTATAAATTGGTGAATCAATTTCCACCACTTAACAATCGCAGATTCTTATTATCTACTCTCTTTGGCAAAATACAAATATTAATATTAGGAATG GrMML10
    TATA-Box         G2-like     |                                                         Start codon
                 Predicted transcription start site
```
