# Supplementary material for: Open-Bud Duplicate Loci Are Identified as MML10s, Orthologs of MIXTA-Like Genes on Homologous Chromosomes of Allotetraploid Cotton
Source: Front Plant Sci. 2020 Feb 18;11:81. doi: 10.3389/fpls.2020.00081 (PMC7040098; doi:10.3389/fpls.2020.00081)
Supplement: Supplementary file 1 [file DataSheet_1.zip › Figure S4.pdf]

Figure S4 Alignment of coding sequences of *MML10*. The primer positions are framed.

```
             10        20        30        40        50        60        70        80        90        100
    ....|....|....|....|....|....|....|....|....|....|....|....|....|....|....|....|....|....|....|....|

                               Start codon
GGCAAAATACAAATATTAATATTAGGAATGGCTGATTGCCGGGAGAAGATGGGGTTGAAGAAAGGGCCATGGACTCCAGATGAAGACCAGAAGCTCTTAG GhMML10_Dt
GGCAAAATACAAATATTAATATTAGGAATGGCTGATTACTGGGAGAAGATGGGGTTGAAGAAAGGGCCATGGACTCCAGATGAAGACCAGAAGCTCTTAG GbMML10_Dt
GGCAAAATACAAATATTAATATTAGGAATGGCTGATTGCCGGGAGAAGATGGGGTTGAAGAAAGGGCCATGGACTCCAGATGAAGACCAGAAGCTCTTAG GhMML10_At
GGCAAAATACAAATATTAATATTAGGAATGGCTGATTACCGGGAGAAGATGGGGTTGAAGAAAGGGCCATGGACTCCAGATGAAGACCAGAAGCTCTTAG GbMML10_At
    Forward primer for qRT-PCR of MML10

            110       120       130       140       150       160       170       180       190       200
    ....|....|....|....|....|....|....|....|....|....|....|....|....|....|....|....|....|....|....|....|
CTTATATTGAAGAACATGGGTTGGGGAATTGGCGTACCTTGCCTGAAAAAGCTGGGCTTCAAAGATGTGGAAAGAGCTGCAGACTGAGATGGATCAATTA GhMML10_Dt
CTTATATTGAAGAACATGGGTTGGGGAATTGGCGTACCTTGCCTGAAAAAGCT-GGCTTCAAAGATGTGGAAAGAGCTGCAGACTGAGATGGATCAATTA GbMML10_Dt
CTTATTTTGAAGAACATGGGCTGGGGAATTGGCGTACCTTGCCTGAAAAAGCTGGGCTTCAAAGATGTGGAAAGAGCTGCAGACTGAGGTGGATCAATTA GhMML10_At
CTTATTTTGAAGAACATGGGCTGGGGAATTGGCGTACCTTGCCTGAAAAAGCTGGGCTTCAAAGATGTGGAAAGAGCTGCAGACTGAGGTGGATCAATTA GbMML10_At
                          Reverse primer for qRT-PCR of MML10

            210       220       230       240       250       260       270       280       290       300
    ....|....|....|....|....|....|....|....|....|....|....|....|....|....|....|....|....|....|....|....|
CCTCAGACCTGATCTCAAGAGGGGAAAATTCAGTTTACAAGAAGAACAAACCATCATCCAACTCCATGCTTTTCTCGGAAACAGGTGGTCAACCATTGCA GhMML10_Dt
CCTCAGACCTGATCTCAAGAGGGGAAAATTCAGTTTACAAGAAGAACAAACCATCATCCAACTCCATGCTTTTCTTGGAAACAGGTGGTCAACCATTGCA GbMML10_Dt
CCTCAGACCTGATCTCAAGAGGGGAAAGTTCAGTTTACAAGAAGAACAAACCATCATCCAACTCCATGCTTTTCTTGGAAACAGGT-------------- GhMML10_At
CCTCAGACCTGATCTCAAGAGGGGAAAGTTCAGTTTACAAGAAGAACAAACCATCATCCAACTCCATGCTTTTCTTGGAAACAGGTGGTCAACCATTGCA GbMML10_At
```

```
         310       320       330       340       350       360       370       380       390       400
....|....|....|....|....|....|....|....|....|....|....|....|....|....|....|....|....|....|....|....|
GCTCACTTACCGAACCGAACCGACAACGAGATTAAAAACTACTGGAACACACATGTTAAGAAACGGTTTACCAAGATGGGGATCGATCCCACCACACACA GhMML10_Dt
GCTCACTTACCGAACCGAACCGACAACGAGATTAAAAACTACTGGAACACACATGTTAAGAAACGGTTTACCAAGATGGGGATCGATCCCACCACACACA GbMML10_Dt
---------------------------------------------------------------------------------------------------- GhMML10_At
GCTCACTTACCGAATCGAACCGACAACGAGATTAAAAACTACTGGAACACACATATTAAGAAACGGTTTACCAAGATGGGGATCGATCCCACCACACACA GbMML10_At

         410       420       430       440       450       460       470       480       490       500
....|....|....|....|....|....|....|....|....|....|....|....|....|....|....|....|....|....|....|....|
AGCCCAAATCGAACCATGTCGTCAGCCCCACCGGTCGAACCACACTGAACCACATGGCTCAATGGGAGAGTGCTAGGCTCGAAGCAGAAGCCAGGCTGGT GhMML10_Dt
AGCCCAAATCGAACCATGTCGTCAGCCCCACCGGTCGAACCACACTGAACCACATGGCTCAATGGGAGAGTGCTAGGCTCGAAGCAGAAGCCAGGCTGGT GbMML10_Dt
---------------------------------------------------------------------------------------------------- GhMML10_At
AGCCCAAATCGAACCATGTCGTCAGCCCCACCGGTCGAACCACAGTGAACCACATGGCTCAATGGGAGAGTGCTAGGCTCGAAGCAGAAGCCAGGCTCGT GbMML10_At

         510       520       530       540       550       560       570       580       590       600
....|....|....|....|....|....|....|....|....|....|....|....|....|....|....|....|....|....|....|....|
CAAAGACTCAAAAAATCTACCCTCATCTTCTTCAAGACCTTCCCCATATCAGAAAAGTTGTAACAAAGGCTCAAAATCCCAGTGCCTTGACGTTGTTAAA GhMML10_Dt
CAAAGACTCAAAAAATCTACCCTCATCTTCTTCAAGACCTTCCCCATATCAGAAAAGTTGTAACAAAGGCTCAAA--------GCGTTGACGTTGTTAAA GbMML10_Dt
---------------------------------------------------------------------------------------------------- GhMML10_At
CAAAGACTCAAAAAAACTACCCTCATCTTCTTCAAGACCTTCCCCATATCAGAAAAGTTGTAACAAAGGCTCAAAATCCCAGTGTCTTGACGTTGTTAAA GbMML10_At

         610       620       630       640       650       660       670       680       690       700
....|....|....|....|....|....|....|....|....|....|....|....|....|....|....|....|....|....|....|....|
GCATGGCAAAGCGTAGTGGCTGGCATGTTCGCCACCTCTACTAACAACTCGAACCGCATCATATTCGGACCAGACCAGAGCTCCGGAAATTACGAGCTTG GhMML10_Dt
GCATGGCAAAGCGTAGTGGCTGGCATGTTCGCCACCTCTACTAACAACTCGAACCGCATCATATTCGGACCAGACCAGAGCTCCGGAAATTACGAGCTTG GbMML10_Dt
```

```
---------------------------------------------------------------------------------------------------- GhMML10_At
GCATGGCAAAGCGTAGTGGCTGGTATGTTCGCCACCTCTACTAACAACTCAAACCGCATCATATTCGGACCAGACCAGAGCTCCGGAAATTACGAGCTTG GbMML10_At

                710       720       730       740       750       760       770       780       790       800
....|....|....|....|....|....|....|....|....|....|....|....|....|....|....|....|....|....|....|....|
ATTCAATTATACCTATTGGAGGTAATGTTGAAGACGAGTTAATGGTAGGCAACGATAGATCAAAGTGCCAGGTACCAGAATTGAATGAAAGGTTTGATAA GhMML10_Dt
ATTCAATTATACCTATTGGAGGTAATGTTGAAGACGAGTTAATGGTAGGCAACGATAGATCAAAGTGCCAGGTACCAGAATTGAATGAAAGGTTTGATAA GbMML10_Dt
---------------------------------------------------------------------------------------------------- GhMML10_At
ATTCAATTATACCTCTTGGAGGTAATGTTGAAGACGAGTTAATGGTAGGCAACGATAGATCAAAGTGCCAGGTACCAGAATTGAATGAAAGGTCTGATAA GbMML10_At

                810       820       830       840       850       860       870       880       890       900
....|....|....|....|....|....|....|....|....|....|....|....|....|....|....|....|....|....|....|....|
TTACATGTCTTTGCATGATACGACGCATCTTTGGGCTGCTCCTATAGCTGAAAACGACGTTGTAGAAGGCTTTCCAGATTTCTTGGTGCATGATTTTGAT GhMML10_Dt
TTACATGTCTTTGCATGATACGACGCATCTTTGGGCTGCTCCTATAGCTGAAAACGACGTTGTAGAAGGCTTTCCAGATTTCTTGGTGCATGATTTTGAT GbMML10_Dt
---------------------------------------------------------------------------------------------------- GhMML10_At
TTACATGTCTTTGCTTGATACGACGCATCTTTGGGCTGCTCCCATAGCTGAAAACGACGTCGTAGAAAGCTTTCCAGATTTCTTGGTGCATGATTTTGAT GbMML10_At

                910       920       930       940
....|....|....|....|....|....|....|....|..
TACCAAATTGACAACGAGGAGTCTATAACCGTTTAA------                                                           GhMML10_Dt
TACCAAATTGACAACGAGGAGTCTATAACCATTTAAAGTTAA                                                           GbMML10_Dt
-------------------------------CTTAA------                                                           GhMML10_At
TACCAAATTGACAACGAGGAGTCTATAACCATTTAA------                                                           GbMML10_At
```
