# Supplementary material for: Open-Bud Duplicate Loci Are Identified as MML10s, Orthologs of MIXTA-Like Genes on Homologous Chromosomes of Allotetraploid Cotton
Source: Front Plant Sci. 2020 Feb 18;11:81. doi: 10.3389/fpls.2020.00081 (PMC7040098; doi:10.3389/fpls.2020.00081)
Supplement: Supplementary file 1 [file DataSheet_1.zip › Figure S5.pdf]

Figure S5 Alignment of the genomic sequences of *MML10* from Gh, Gb, *G. arboreum* and *G. raimondii*. Letters with yellow background: exon region.

```
         10        20        30        40        50        60        70        80        90        100
....|....|....|....|....|....|....|....|....|....|....|....|....|....|....|....|....|....|....|....|
ATGGCTGATTGCCGGGAGAAGATGGGGTTGAAGAAAGGGCCATGGACTCCAGATGAAGACCAGAAGCTCTTAGCTTATTTTGAAGAACATGGGCTGGGGA GhMML10_At(TM-1)
ATGGCTGATTGCCGGGAGAAGATGGGGTTGAAGAAAGGGCCATGGACTCCAGATGAAGACCAGAAGCTCTTAGCTTATATTGAAGAACATGGGTTGGGGA GhMML10_Dt(TM-1)
ATGGCTGATTGCCGGGAGAAGATGGGGTTGAAGAAAGGGCCATGGACTCCAGATGAAGACCAGAAGCTCTTAGCTTATATTGAAGAACATGGGTTGGGGA GbMML10_Dt(3-79)
ATGGCTGATTACCGGGAGAAGATGGGGTTGAAGAAAGGGCCATGGACTCCAGATGAAGACCAGAAGCTCTTAGCTTATTTTGAAGAACATGGGCTGGGGA GbMML10_At(3-79)
ATGGCTGATTGCCGGGAGAAGATGGGGTTGAAGAAAGGGCCATGGACTCCAGATGAAGACCAGAAGCTCTTAGCTTATATTGAAGAACATGGGTTGGGGA GrMML10
ATGGCTGATTGCCGGGAGAAGATGGGGTTGAAGAAAGGGCCATGGACTCCAGATGAAGACCAGAAGCTCTTAGCTTATTTTGAAGAACATGGGCTGGGGA GaMML10(Shixiya1)

        110       120       130       140       150       160       170       180       190       200
....|....|....|....|....|....|....|....|....|....|....|....|....|....|....|....|....|....|....|....|
ATTGGCGTACCTTGCCTGAAAAAGCTGGTGACTATCCATCATCTTTTTTTTCCATTTTAGTATTCTGCAAATACGCATTCTTTCTATACGTTTTGCTGGT GhMML10_At(TM-1)
ATTGGCGTACCTTGCCTGAAAAAGCTGGTGACCATCCATCATCTTTTTT--CCATTTTAGTATTCAGCAAATACGCGTTCTTTCTATACGTTTTGCTGGT GhMML10_Dt(TM-1)
ATTGGCGTACCTTGCCTGAAAAAGCTGGTGACCATCCATCATCTTTTTT--CCATTTTAGTATTCAGCAAATACGCATTCTTTGTATACGTTTTGCTGGT GbMML10_Dt(3-79)
ATTGGCGTACCTTGCCTGAAAAAGCTGGTGACTATCCATCATCTTTTTTTTCCATTTTAGTATTCTGCAAATACGCATTCTTTCTATACGTTTTGCTGGT GbMML10_At(3-79)
ATTGGCGTACCTTTCCTGAAAAAGCTGGTGACCGTCCATCATCTTTTTT--CCATTTTAGTATTCAGCAAATACGCATTCTTTCTATACGTTTTGCTGGT GrMML10
ATTGGCGTACCTTGCCTGAAAAAGCTGGTGACTATCCATCATCTTTTTTTTCCATTTTAGTATTCTGCAAATACGCATTCTTTCTATACGTTTTGCTGGT GaMML10(Shixiya1)

        210       220       230       240       250       260       270       280       290       300
....|....|....|....|....|....|....|....|....|....|....|....|....|....|....|....|....|....|....|....|
CTACTTTATAACTGACCAAAATGCTTCTTCTTTTTATCTCAAATAATAATAATAAATAAATAAATAAGGGCTTCAAAGATGTGGAAAGAGCTGCAGACTG GhMML10_At(TM-1)
CTACTTTATAACTGACCAAAATGCTTCTTCTTTTTATCTCAAATAATAACAAAAAATAAG--------GGCTTCAAAGATGTGGAAAGAGCTGCAGACTG GhMML10_Dt(TM-1)
CTACTTTATAACTGACCAAAATGCTTCTTCTTTTTATCTCAAATAATAACAATAAATAAA--------GGCTTCAAAGATGTGGAAAGAGCTGCAGACTG GbMML10_Dt(3-79)
CTACTTTATAACTGACCAAAATGCTTCTTCTTTTTATCTCAAATAATAATAATAAATAAATAAATAAGGGCTTCAAAGATGTGGAAAGAGCTGCAGACTG GbMML10_At(3-79)
```

```
CTACTTTATAACTGACCAAAATGCTTCTTCTTTTTATCTCAAATAATAATAATAAATAAG--------GGCTTCAAAGATGTGGAAAGAGCTGCAGACTG GrMML10
CTACTTTATAACTGACCAAAATGCTTCTTCTTTTTATCTCAAATAATAATAATAAATAAATAAATAAGGGCTTCAAAGATGTGGAAAGAGCTGCAGACTG GaMML10(Shixiya1)

        310       320       330       340       350       360       370       380       390       400
....|....|....|....|....|....|....|....|....|....|....|....|....|....|....|....|....|....|....|....|
AGGTGGATCAATTACCTCAGACCTGATCTCAAGAGGGGAAAGTTCAGTTTACAAGAAGAACAAACCATCATCCAACTCCATGCTTTTCTTGGAAACAGGT GhMML10_At(TM-1)
AGATGGATCAATTACCTCAGACCTGATCTCAAGAGGGGAAAATTCAGTTTACAAGAAGAACAAACCATCATCCAACTCCATGCTTTTCTCGGAAACAGGT GhMML10_Dt(TM-1)
AGATGGATCAATTACCTCAGACCTGATCTCAAGAGGGGAAAATTCAGTTTACAAGAAGAACAAACCATCATCCAACTCCATGCTTTTCTTGGAAACAGGT GbMML10_Dt(3-79)
AGGTGGATCAATTACCTCAGACCTGATCTCAAGAGGGGAAAGTTCAGTTTACAAGAAGAACAAACCATCATCCAACTCCATGCTTTTCTTGGAAACAGGT GbMML10_At(3-79)
AGATGGATCAATTACCTCAGACCTGATCTCAAGAGGGGAAAATTCAGTTTACAAGAAGAACAAACCATCATCCAACTCCATGCTTTTCTTGGAAACAGGT GrMML10
AGGTGGATCAATTACCTCAGACCTGATCTCAAGAGGGGAAAGTTCAGTTTACAAGAAGAACAAACCATCATCCAACTCCATGCTTTTCTTGGAAACAGGT GaMML10(Shixiya1)

        410       420       430       440       450       460       470       480       490       500
....|....|....|....|....|....|....|....|....|....|....|....|....|....|....|....|....|....|....|....|
CTTAAGATGCCACTTCTCCACTTCTCTCCCTAAACCATCAAATCTTGTAAATACCCTTTAATTAAAACAAATTCAACAGTATGGTGGAATCAATCAAATC GhMML10_At(TM-1)
CTTAAGATGCCACTTCTCCACTTCTCTCCCTAAACCATCAAATCTTTTAAATACCCTTTAATTAAAACAAATTCAACAGTATGGTGGAATCAATCAAATC GhMML10_Dt(TM-1)
CTTAAGATGCCACTTCTCCACTTCTCTCCCTAAACCATCAAATCTTTTAAATACCCTTTAATTAAAACAAATTCAACAGTATGGTGGAATCAATCAAATC GbMML10_Dt(3-79)
CTTAAGATGCCACTTCTCCACTTCTCTCCCTAAACCATCAAATCTTGTAAATACCCTTTAATTAAAACAAATTCAACAGTATGGTGGAATCAATCAAATC GbMML10_At(3-79)
CTTAAGATGCCACTTCTCCACTTCTCTCCCTAAGCCATCAAATCTTTTAAATACCCTTTAATTAAAACAAATTCAACAGTATGGTGGAATCAATCAAATC GrMML10
CTTAAGATGCCACTTCTCCACTTCTCTCCCTAAACCATCAAATCTTGTAAATACCCTTTAATTAAAACAAATTCAACAGTATGGTGGAATCAATCAAATC GaMML10(Shixiya1)

        510       520       530       540       550       560       570       580       590       600
....|....|....|....|....|....|....|....|....|....|....|....|....|....|....|....|....|....|....|....|
TGGGTTTTAAGGATTTCAAAGAATAACAATGGAGTGAAACAAGATAGACCCACAGTT-GTTATATTCTATTCCACTCTACTATGAAGGTGTAATTTTGAA GhMML10_At(TM-1)
TGGGTTTTAAGGATTTCAAAGAATAACAATGGAGTGAAACGAGATTGACCCACAGTTTGTTATATTCTATTCCACTCCATTATGAAGGTGTAATTTTGAA GhMML10_Dt(TM-1)
TGGGTTTTAAGGATTTCAAAGAATAACAATGGAGTGAAACGAGATTGACCCACAGTTTGTTATATTCTATTCCACTCCATTATGAAGGTGTAATTTTGAA GbMML10_Dt(3-79)
TGGGTTTTAAGGATTTCAAAGAATAACAATGGAGTGAAACAAGATAGACCCACAGTT-GTTATATTCTATTCCACTCTACTATGAAGGTGTAATTTTGAA GbMML10_At(3-79)
```

```
TGGGTTTTAAGGATTTCAAAGAATAACAATGGAGTGAAACGAGATAGACCCACAGTTTGTTATATTCTATTCCACTCCATTATGAAGGTGTAATTTTGAA GrMML10
TGGGTTTTAAGGATTTCAAAGAATAACAATGGAGTGAAACAAGATAGACCCACAGTT-GTTATATTCTATTCCACTCTACTGTGAAGGTGTAATTTTGAA GaMML10(Shixiya1)

                 610       620       630       640       650       660       670       680       690       700
        ....|....|....|....|....|....|....|....|....|....|....|....|....|....|....|....|....|....|....|....|
AACTCTTATATATTTCCATGGATGTTGGAACATCTATCTTTATTGTTCAAAAAGAAATATATCCTTGAGACTTGCCCAATCATTTTAAATTTTTTGAACT GhMML10_At(TM-1)
AACTCTTATATATTTCCATGGATGTTGGAACATCTATCTTTATTGTTCAAAAAGAAATCTATCCTTGAGACTTGCTCAATCATTTTAAATTTTTTGAACT GhMML10_Dt(TM-1)
AACTCTTATATATTTCCATGGATGTTGGAACATCTATCTTTATTGTTCAAAAAGAAATCTATCCTTGAGACTTGCTCAATCATTTTAAATTTTTTGAACT GbMML10_Dt(3-79)
AACTCTTATATATTTCCATGGATGTTGGAACATCTATCTTTATTGTTCAAAAAGAAATATATCCTTGAGACTTGCCCAATCATTTTAAATTTTTTGAACT GbMML10_At(3-79)
AACTCTTATATATTTCCATGGATGTTGGAACATCTATCTTTATTGTTCAAAAAGAAATCTATCCTTGAGACTTGCTCAATCATTTTAAATTTTTTGAACT GrMML10
AACTCTTATATATTTCCATGGATGTTGGAACATCTATCTTTATTGTTCAAAAAGAAATCTATCCTTGAGACTTGCCCAATCATTTTAAATTTTTTGAACT GaMML10(Shixiya1)

                 710       720       730       740       750       760       770       780       790       800
        ....|....|....|....|....|....|....|....|....|....|....|....|....|....|....|....|....|....|....|....|
AATTTTTTATTAGTTTCAATTATTATTAAATCGAGTTAAGTTCAAATTTATGTGCATGTCTAAATCAAGTACATTATAGTTTATATTATATTTGGGTATT GhMML10_At(TM-1)
AATTTTTTATTAGCTTCAATTATTATTAAATCGATTTAAGTTCAAATTTATGTGCATGTCTAAATCAAGTACATTATAGTTTATATTATATTTGGGTATT GhMML10_Dt(TM-1)
AATTTTTTATTAGCTTCAATTATTATTAAATCGATTTAAGTTCAAATTTATGTGCATGTCTAAATCAAGTACATTATAGTTTATATTATATTTGGGTATT GbMML10_Dt(3-79)
AATTTTTTATTAGTTTCAATTATTATTAAATCGAGTTAAGTTCAAATTTATGTGCATGTCTAAATCAAGTACATTATAGTTTATATTATATTTGGGTATT GbMML10_At(3-79)
AATTTTTTATTAGCTTCAATTATTATTAAATCGATTTAAGTTCAAATTTATGTGCATGTCTAAATCAAGTACATTATAGTTTATATTATATTTGGGTATT GrMML10
AATTTTTTATTAGTTTCAATTATTATTAAATCGAGTTAAGTTGAAATTTATGTGCATCTCTAAATCAAGTACATTATAGTTTATATTATATTTGGGTATT GaMML10(Shixiya1)

                 810       820       830       840       850       860       870       880       890       900
        ....|....|....|....|....|....|....|....|....|....|....|....|....|....|....|....|....|....|....|....|
TCAGCTTCAAGCCATTCGTGTA------------------------------------------------------------------------------ GhMML10_At(TM-1)
TCAGCTTCAAGCCATTCGTGTAAAGTTTA--CACTATTTACTACTGG--TACTGGACTGTAAATTTAAGCATAGGGAAGTTTGTTGTTGCATGCATGCAA GhMML10_Dt(TM-1)
TCAGCTTCAAGCCATTCGTGTAAAGTTTA--CACTATTTACTACTGG--TACTGGACTGTAAATTTAAGCATAGGGAAGTTTGTTGTTGCATGCATGCAA GbMML10_Dt(3-79)
TCAGCTTCAAGCCATTCGTGTATAGTTTA--CACTATTTACTACTGG--TACTGGACTGTAAATTTAAGCATAGGGAAATTTGCTGTTGCATGCATGCAA GbMML10_At(3-79)
```

```
TCAGCTTCAAGCCATTCGTGTAAAGTTTA--CACTATTTACTACTGG--TACTGGACTGTAAATTTAAGCATAGGGAAGTTTGTTGTTGCATGCATGCAA GrMML10
TCAGCTTCAAGCCATTCGTGTATAGTTTA--CACTATTTACTACTGG--TACTGGACTGTAAATTTAAGCATAGGGAAATTTGCTGTTGCATGCATGCAA GaMML10(Shixiya1)

                910       920       930       940       950       960       970       980       990      1000
        ....|....|....|....|....|....|....|....|....|....|....|....|....|....|....|....|....|....|....|....|
        ---------------------------------------------------------------------------------------------------- GhMML10_At(TM-1)
        TTATTTCCAATT----------TCAGATTATTTACAAAATTCTACGTACCTTTCCTGTGAAGGTGGTCAACCATTGCAGCTCACTTACCGAACCGAACCG GhMML10_Dt(TM-1)
        TTATTTCCAATT----------TCAGATTATTTACAAAATTCTATGTACCTTTCCTGTGAAGGTGGTCAACCATTGCAGCTCACTTACCGAACCGAACCG GbMML10_Dt(3-79)
        TTATTTCCAATT----------TCAGATTATTTACAAAATTCTACGTACCTTTCCTGTGAAGGTGGTCAACCATTGCAGCTCACTTACCGAATCGAACCG GbMML10_At(3-79)
        TTATTTCCAATTATTTCCAATTTCAGATTATTTACAAAATTCTACGTACCTTTCCTGTGAAGGTGGTCAACCATTGCAGCTCACTTACCGAACCGAACCG GrMML10
        TTATTTCCAATT----------TCAGATTATTTACAAAATTCAACGTACCTTTCCTGTGAAGGTGGTCAACCATTGCAGCTCACTTACCGAATCGAACCG GaMML10(Shixiya1)

               1010      1020      1030      1040      1050      1060      1070      1080      1090      1100
        ....|....|....|....|....|....|....|....|....|....|....|....|....|....|....|....|....|....|....|....|
        ---------------------------------------------------------------------------------------------------- GhMML10_At(TM-1)
        ACAACGAGATTAAAAACTACTGGAACACACATGTTAAGAAACGGTTTACCAAGATGGGGATCGATCCCACCACACACAAGCCCAAATCGAACCATGTCGT GhMML10_Dt(TM-1)
        ACAACGAGATTAAAAACTACTGGAACACACATGTTAAGAAACGGTTTACCAAGATGGGGATCGATCCCACCACACACAAGCCCAAATCGAACCATGTCGT GbMML10_Dt(3-79)
        ACAACGAGATTAAAAACTACTGGAACACACATATTAAGAAACGGTTTACCAAGATGGGGATCGATCCCACCACACACAAGCCCAAATCGAACCATGTCGT GbMML10_At(3-79)
        ACAACGAGATTAAAAACTACTGGAACACACATGTTAAGAAACGGTTTACCAAGATGGGGATCGATCCCACCACACACAAGCCCAAATCGAACCATGTCGT GrMML10
        ACAACGAGATTAAAAACTACTGGAACACACATATTAAGAAACGGTTTACCAAGATGGGGATCGATCCCACCACACACAAGCCCAAATCGAACCATGTCGT GaMML10(Shixiya1)

               1110      1120      1130      1140      1150      1160      1170      1180      1190      1200
        ....|....|....|....|....|....|....|....|....|....|....|....|....|....|....|....|....|....|....|....|
        ---------------------------------------------------------------------------------------------------- GhMML10_At(TM-1)
        CAGCCCCACCGGTCGAACCACACTGAACCACATGGCTCAATGGGAGAGTGCTAGGCTCGAAGCAGAAGCCAGGCTGGTCAAAGACTCAAAAAATCTACCC GhMML10_Dt(TM-1)
        CAGCCCCACCGGTCGAACCACACTGAACCACATGGCTCAATGGGAGAGTGCTAGGCTCGAAGCAGAAGCCAGGCTGGTCAAAGACTCAAAAAATCTACCC GbMML10_Dt(3-79)
        CAGCCCCACCGGTCGAACCACAGTGAACCACATGGCTCAATGGGAGAGTGCTAGGCTCGAAGCAGAAGCCAGGCTCGTCAAAGACTCAAAAAAACTACCC GbMML10_At(3-79)
```

```
CAGCCCCACCGGTCGAACCACACTGAACCACATGGCTCAATGGGAGAGTGCTAGGCTCGAAGCAGAAGCCAGGCTGGTCAAAGACTCAAAAAATCTACCC GrMML10
CAACCCCACCGGTCGAACCACAGTGAACCACATGGCTCAATGGGAGAGTGCTAGGCTCGAAGCAGAAGCCAGGCTCGTCAAAGACTCAAAAAAACTACCC GaMML10(Shixiya1)

               1210      1220      1230      1240      1250      1260      1270      1280      1290      1300
       ....|....|....|....|....|....|....|....|....|....|....|....|....|....|....|....|....|....|....|....|
----------------------------------------------------------------------------------------------------- GhMML10_At(TM-1)
TCATCTTCTTCAAGACCTTCCCCATATCAGAAAAGTTGTAACAAAGGCTCAAAATCCCAGTGCCTTGACGTTGTTAAAGCATGGCAAAGCGTAGTGGCTG GhMML10_Dt(TM-1)
TCATCTTCTTCAAGACCTTCCCCATATCAGAAAAGTTGTAACAAAGGCTCAAA--------GCGTTGACGTTGTTAAAGCATGGCAAAGCGTAGTGGCTG GbMML10_Dt(3-79)
TCATCTTCTTCAAGACCTTCCCCATATCAGAAAAGTTGTAACAAAGGCTCAAAATCCCAGTGTCTTGACGTTGTTAAAGCATGGCAAAGCGTAGTGGCTG GbMML10_At(3-79)
TCATCTTCTTCAAGACCTTCCCCATATCAGAAAAGTTGTAACAAAGGCTCAAAATCCCAGTGCCTTGACGTTGTTAAAGCATGGCAAAGCGTAGTGGCTG GrMML10
TCATCTTCTTCAAGACCTTCCCCATATCAGAAAAGTTGTAACAAAGGCTCAAAATCCCAGTGTCTTGACGTTGTTAAAGCATGGCAAAGCGTAGTGGCTG GaMML10(Shixiya1)

               1310      1320      1330      1340      1350      1360      1370      1380      1390      1400
       ....|....|....|....|....|....|....|....|....|....|....|....|....|....|....|....|....|....|....|....|
----------------------------------------------------------------------------------------------------- GhMML10_At(TM-1)
GCATGTTCGCCACCTCTACTAACAACTCGAACCGCATCATATTCGGACCAGACCAGAGCTCCGGAAATTACGAGCTTGATTCAATTATACCTATTGGAGG GhMML10_Dt(TM-1)
GCATGTTCGCCACCTCTACTAACAACTCGAACCGCATCATATTCGGACCAGACCAGAGCTCCGGAAATTACGAGCTTGATTCAATTATACCTATTGGAGG GbMML10_Dt(3-79)
GTATGTTCGCCACCTCTACTAACAACTCAAACCGCATCATATTCGGACCAGACCAGAGCTCCGGAAATTACGAGCTTGATTCAATTATACCTCTTGGAGG GbMML10_At(3-79)
GCATGTTCGCCACCTCTACTAACAACTCGAACCGCATCATATTCGGACCAGACCAGAGCTCCGGAAATTACGAGCTTGATTCAATTATACCTATTGGAGG GrMML10
GTATGTTCGCCACCTCTACTAACAACTCAAACCGCATCATATTCGGACCAGACCAGAGCTCCGGAAATTACGAGCTTGATTCAATTATACCTCTTGGAGG GaMML10(Shixiya1)

               1410      1420      1430      1440      1450      1460      1470      1480      1490      1500
       ....|....|....|....|....|....|....|....|....|....|....|....|....|....|....|....|....|....|....|....|
----------------------------------------------------------------------------------------------------- GhMML10_At(TM-1)
TAATGTTGAAGACGAGTTAATGGTAGGCAACGATAGATCAAAGTGCCAGGTACCAGAATTGAATGAAAGGTTTGATAATTACATGTCTTTGCATGATACG GhMML10_Dt(TM-1)
TAATGTTGAAGACGAGTTAATGGTAGGCAACGATAGATCAAAGTGCCAGGTACCAGAATTGAATGAAAGGTTTGATAATTACATGTCTTTGCATGATACG GbMML10_Dt(3-79)
TAATGTTGAAGACGAGTTAATGGTAGGCAACGATAGATCAAAGTGCCAGGTACCAGAATTGAATGAAAGGTCTGATAATTACATGTCTTTGCTTGATACG GbMML10_At(3-79)
```

```
TAATGTTGAAGACGAGTTAATGGTAGGCAACGATAGATCAAAGTGCCAGGTACCAGAATTGAATGAAAGGTTTGATAATTACATGTCTTTGCATGATACG GrMML10
TAATGTTGAAGACGAGTTAATGGTAGGCAACGATAGATCAAAGTGCCAGGTACCAGAATTGAATGAAAGGTTTGATAATTACATGTCTTTGCATGATACG GaMML10(Shixiya1)

               1510      1520      1530      1540      1550      1560      1570      1580      1590      1600
       ....|....|....|....|....|....|....|....|....|....|....|....|....|....|....|....|....|....|....|....|
----------------------------------------------------------------------------------------------------- GhMML10_At(TM-1)
ACGCATCTTTGGGCTGCTCCTATAGCTGAAAACGACGTTGTAGAAGGCTTTCCAGATTTCTTGGTGCATGATTTTGATTACCAAATTGACAACGAGGAGT GhMML10_Dt(TM-1)
ACGCATCTTTGGGCTGCTCCTATAGCTGAAAACGACGTTGTAGAAGGCTTTCCAGATTTCTTGGTGCATGATTTTGATTACCAAATTGACAACGAGGAGT GbMML10_Dt(3-79)
ACGCATCTTTGGGCTGCTCCCATAGCTGAAAACGACGTCGTAGAAAGCTTTCCAGATTTCTTGGTGCATGATTTTGATTACCAAATTGACAACGAGGAGT GbMML10_At(3-79)
ACGCATCTTTGGGCTGCTCCTATAGCTGAAAACGACGTTGTAGAAGGCTTTCCAGATTTCTTGGTGCATGATTTTGATTACCAAATTGACAACGAGGAGT GrMML10
ACGCATCTTTGGGCTGCTCCCATAGCTGAAAACGACGTCGTAGAAAGCTTTCCAGATTTCTTGGTGCATGATTTTGATTACCAAATTGACAACGAGGAGT GaMML10(Shixiya1)

               1610      1620      1630      1640      1650      1660      1670      1680      1690      1700
       ....|....|....|....|....|....|....|....|....|....|....|....|....|....|....|....|....|....|....|....|
----------------------------------------------------------------------------------------------------- GhMML10_At(TM-1)
CTATAACCGTTTAAAAAATTTATTTT-------------------GGTATTTGAATTTGGTAATTAATTTTATTTCTTCTAAATTTGGATTTCATCAAGA GhMML10_Dt(TM-1)
CTATAACCGTTTAAAAAATTTATTTT-------------------GGTATTTGAATTTGGTAATTAATTTTATTTCCTCTAAATTTGGATTTCATCAAGA GbMML10_Dt(3-79)
CTATAACCATTTAAAAAATTCATTTTAGTATTTTTTT-ACATTTTGGTATTTAAATTTGGTAATTAATTT--TTTTCTCTAAACTTGAATTTTGTCAAGA GbMML10_At(3-79)
CTATAACCGTTTAAAAAATTCATTTTGGTATTTTTTTTATATTTTGGTATTTGAATTTGGTAATTAATTTTATTTCCTCTAAATTTGGATTTCGTCAAGA GrMML10
CTATAACCATTTAAAAAATTCATTTTCGTATTTTTTT-ACATTTTGGTATTTAAATTTGGTAATTAATTT--TTTTCTCTAAACTTGAATTTTGTCAAGA GaMML10(Shixiya1)

               1710      1720      1730      1740      1750      1760      1770      1780      1790      1800
       ....|....|....|....|....|....|....|....|....|....|....|....|....|....|....|....|....|....|....|....|
----------------------------------------------------------------------------------------------------- GhMML10_At(TM-1)
TTTGATGATATGGTACTCTTGACATCTTTGAACTTTTATATTTTTTAAGTTTAAAGATCAAAATAAATCTAATTATTAAGTTCAAGTACTAAAATAAACA GhMML10_Dt(TM-1)
TTTGATGATATGGTACTCTTGACATCTTTGAACTTTTATATTTTTTAAGTTTAAAGATCAAAATAAATCTAATTATTAAGTTCAAGTACTAAAATAAACA GbMML10_Dt(3-79)
TTTGATGATATGATACTCTTGACATCTTTAAATTTTTATATTTTTTAAGTTCAAAGATCAAAATAAACCTAATTATTAAGTTTAAGTACTAAA-TAAACA GbMML10_At(3-79)
```

```
TTTGATGATATGGTACTCTTGACATCTTTGAACTTTTATATTTTTTAAGTTTAAAGATCAAAATAAATCTAATTATTAAGTTCAAGTACTAAAATAAACA GrMML10
TTTGATGATATGATACTCTTGACATCTTTAAATTTT-ATATTTTT-AAGTTCAAAGATCAAAATAAACCTAATTATTAAGTTTAAGTACTAAAGTAAACA GaMML10(Shixiya1)

              1810      1820      1830      1840      1850      1860      1870      1880      1890      1900
....|....|....|....|....|....|....|....|....|....|....|....|....|....|....|....|....|....|....|....|
---------------------------------------------------------------------------------------------------- GhMML10_At(TM-1)
CATACAAAAAATTATAAATATCAAAAT-ACCTAATTATCGAATTGAAAATTTGAAAATTAGATTAACTCTTAACAAGATATCAAATATAGGAAATGTAAT GhMML10_Dt(TM-1)
CATACAAAAAATTATAAATATCAAAAT-ACCTAATTATCGAATTGAAAATTTGAAAATTAGATGAACTCTTAACCAGATATCAAATATAGGAAATGTAAT GbMML10_Dt(3-79)
--TAAAAACAATTATAAATATCAAAATTATCTAATTATCGAATTCAAAATTTAAAAATTGGATTAACTCTTAACAAGATATCAAACATAGGAAATGTCAT GbMML10_At(3-79)
CATACAAAAAATTATAAATATCAAAATGACCTAATTATCGAATTCAAAATTTGAAAATTAGATTAACTCTTAACAAGATATCAAATATAGGAAATGTAAT GrMML10
--TAAAAACAATTATAAATATCAAAATTATCTAATTATCGAATTCAAAATTTAAAAATTGGATTAACTCTTAACAAGATATCAAATATAGGAAATGTCAT GaMML10(Shixiya1)

              1910      1920      1930      1940      1950      1960      1970      1980      1990      2000
....|....|....|....|....|....|....|....|....|....|....|....|....|....|....|....|....|....|....|....|
---------------------------------------------------------------------------------------------------- GhMML10_At(TM-1)
GTAATGGGAGATCAATGTTGGAGAAGTAAATTAATTCCATTTGTGCTATATTTGGTTATTACTATTTTACTTCATCAAGAGATTATTAGCAAAGTCTTAC GhMML10_Dt(TM-1)
GTAATGGGAGATCAATGTTGGAGAAGTAAATTAATTCCATTTGTGCTATATTTGGTTATTACTATTTTACTTCATCAAGAGATTATTAGCAAAGTCTTAT GbMML10_Dt(3-79)
GTAATGGGAGATCAACGTTGGAGAAGTAAATTAATTCCATTTGTGCTATATTTGGTTATTACTATTTCACTTTATCAAGAGATTATTAGCGAAATCTTAT GbMML10_At(3-79)
GCAATGGGAGATCAATGTTGGAGAAGTAAATTAATTCCATTTGTGCTATATTTGGTTTTTACTATTTTACTTCATCAAGAGATTATTAGCAAAGTCTTAT GrMML10
GTAATGGGAGATCAACGTTGGAGAAGTAAATTAATTCCATTTGTGCTATATTTGGTTATTACTATTTTACTTTATCAAGAGATTATTAGCGAAATCTTAT GaMML10(Shixiya1)

              2010      2020      2030      2040      2050      2060      2070      2080      2090      2100
....|....|....|....|....|....|....|....|....|....|....|....|....|....|....|....|....|....|....|....|
---------------------------------------------------------------------------------------------------- GhMML10_At(TM-1)
TTAAAAATAAAAAATAAAATATGAGTTTTTTTT--ATAGTATGAGATTATATCAATTGATTGAACTTAAAATTAATGGTCTAATCAGTTTAAATAATACT GhMML10_Dt(TM-1)
TTAAAAATAAAAAATAAAATATGAGTTTTTTTTT-ATAGTATGAGATTATATCAATTGATTGAACTTAAAATTAATGGTCTAATCAGTTTAAATAATACT GbMML10_Dt(3-79)
TAATAAAAAATATATATAATATGAGTTTTTTTTTTATAGTATGAGATTATATCAATTGGTTCAATTTAAAATTAATGGTCTAATCAGTTTAAATAATATT GbMML10_At(3-79)
```

```
TTAAAAATAAAAAATAAAATATGAGTTTTTTTTTTATAGTATGAGATTACATCAATTGATTGAACTTAAAATTAATGGTCGAATCAGTTTAAATAATACT GrMML10
TAATAAAAAATATATATAATATGAGTTTTTTTTT-ATAGTATGAGATTATATCAATTGGTTTAATTTAAAATTAATGGTCTAATCAGTTTAAATAATATT GaMML10(Shixiya1)

               2110      2120      2130      2140      2150      2160      2170      2180      2190      2200
....|....|....|....|....|....|....|....|....|....|....|....|....|....|....|....|....|....|....|....|
---------------------------------------------------------------------------------------------------- GhMML10_At(TM-1)
ATAAAATAGAACAAAGGGTAAAATAATCGTTGAAGCGACAACAAAATTACTCAAACCAGATAAAAATTGGTCGAATCAAATTTAAGTTAGTTTAAATATT GhMML10_Dt(TM-1)
ATAAAATAGAACAAAGGGTAAAATAATCGTTGAAGCGACAACAAAATTACTCAAACCAGATAAAAATTGGTCGAATCAAATTTAAGTTAGTTTAAATATT GbMML10_Dt(3-79)
ATAAAACAGAGCAAGGGGTAAAATAATCGTTCAAGCGACAACAAAATTAATCAAACAAGATAAAAATTGGTCAAATCAAATTTAAGTTAGTTTAAATATT GbMML10_At(3-79)
ATAAAATAGAACAAGGAGTAAAATAATCGTTGAAGCGACAATAAAATTACTCAAACCAGATAAAAATTGGTCGAATCAAATTAAAGTTAGTTTAAATATT GrMML10
ATAAAACAGAGCAAGGGGTAAAATAATCGTTCAAGCGACAACAAAATTAATCAAACAAGATAAAAATTGGTCGAATCAAATTTAAGTTAGTTTAAATATT GaMML10(Shixiya1)

               2210      2220      2230      2240      2250      2260      2270      2280      2290      2300
....|....|....|....|....|....|....|....|....|....|....|....|....|....|....|....|....|....|....|....|
---------------------------------------------------------------------------------------------------- GhMML10_At(TM-1)
CAATTTTGTTGTTATATATTTTTTTTTAGAAAAAATGGATGTTTATGTTTTTTATTATTCATTTGATTTTTGTTTTTAAATACTTTAGTTTGCTTTTA-TA GhMML10_Dt(TM-1)
CAATTTTGTTGTTATATTTTTTTTTTTAGAAAAAATGGATGTTTATGTTTTTTATTATTCATTTGATTTTTGTTTTTAAATACTTTAGTTTGCTTTTA-TA GbMML10_Dt(3-79)
CAATTTTGTTGTTATATATTTATTTTTAGAAAAATGGATGTTTATGTTTTTTGTTATTCATTTGATTTTTGTTTTTAAATATTTTAGTTTGTTTTTA-TA GbMML10_At(3-79)
CAATTTTGTTGTTATAT--TTTTTTTAGAAAAAATGGATGTTTATGTTTTTTATTATTCA--------------------------------------- GrMML10
CAATTTTGTTGTTATATATTTATTTTTAGAAAAATGGATGTTTATGTTTTTTGTTATTCATTTGATTTTTGTTTTTAAATATTTTAGTTTGTTTTTA-TA GaMML10(Shixiya1)

               2310      2320      2330      2340      2350      2360      2370      2380      2390      2400
....|....|....|....|....|....|....|....|....|....|....|....|....|....|....|....|....|....|....|....|
---------------------------------------------------------------------------------------------------- GhMML10_At(TM-1)
CTTCATTAAATATTTAATTTTTTACTGTAATTTTTATGAATTTTTATATTTAAAAAAAATTATTTTGTAATGGTTGAACTGAAAGTCAATGTTCTTATCA GhMML10_Dt(TM-1)
CTTCATTAAATATTTAATTTTTTATTGTAATTTTTATGAATTTTTATATTTAAAAAAAATTATTTTGTAATGGTTGAACTGAAAGTCAATGTTCTTATCA GbMML10_Dt(3-79)
TTTCATTAAATATTTAATTTTTTATTGTAATTCTTATGAATTTGTATATTTTAAAAATATTATTATGTAATGGTTGAACTGAAAGTCAATGTTTTTATCA GbMML10_At(3-79)
```

```
---------------------------------------------------------------------------------------------------- GrMML10
TTTCATTAAATATTTAATTTTTTATTGTAATTCTTATGAATTTGTATATTTTAAAAATATTATTATTTAATGGTTGAACTGAAAGTCAATGTTTTTATCA GaMML10(Shixiya1)

              2410      2420      2430      2440      2450      2460      2470      2480      2490      2500
....|....|....|....|....|....|....|....|....|....|....|....|....|....|....|....|....|....|....|....|
---------------------------------------------------------------------------------------------------- GhMML10_At(TM-1)
ATTTGAC--------------------------------------------------------------------------------------------- GhMML10_Dt(TM-1)
ATTTGAC--------------------------------------------------------------------------------------------- GbMML10_Dt(3-79)
ATTCTACCATTGTTTTGGTTCTGCATTTTTGTGTCAATGAGTAAAATATAAATTTAATTTTGAAATTCACCCTTTATATTATAAAAATTGAGAACTTAAT GbMML10_At(3-79)
---------------------------------------------------------------------------------------------------- GrMML10
ATTCGACCATTGGTTTGGTTCTGCATTTTTGTGTCAATGAGTAAAATATAAATTTAATTTTGAAATTCACCCTTTATATTATAAAAATTGAGAACTTAAT GaMML10(Shixiya1)

              2510      2520      2530      2540      2550      2560      2570      2580      2590      2600
....|....|....|....|....|....|....|....|....|....|....|....|....|....|....|....|....|....|....|....|
---------------------------------------------------------------------------------------------------- GhMML10_At(TM-1)
---------------------------------------------------------------------------------------------------- GhMML10_Dt(TM-1)
---------------------------------------------------------------------------------------------------- GbMML10_Dt(3-79)
ATCTTTACCTTAATTTATCGAAATTTGATCCTCTTATCTTACGAAAAAATGGAAAGTTAATCCAATTGTTGGTAAATATATGAATTTGAATGGCTAATTT GbMML10_At(3-79)
---------------------------------------------------------------------------------------------------- GrMML10
ATCTTTACCTTAATTTATCGAAATTTGATCCTCTTATCTTACGAAAAAATGGAAAGTTAATCCAATTGTTGGTAAACATATGAATTTGAATGACTAATTT GaMML10(Shixiya1)

              2610      2620      2630      2640      2650      2660      2670      2680      2690      2700
....|....|....|....|....|....|....|....|....|....|....|....|....|....|....|....|....|....|....|....|
--------------------------ATTTTTATTCATTATTTACATATAAATTGTTGAACTATAAATTTTTAAATTAGATATTTAACTGAAAATTTTAA GhMML10_At(TM-1)
------------------AAGCGTTAATTTTTATTCATTATTTACATATAAATTTTTGAACTAGTAATTTTTAAATCAGATATTTAACTGAAAATTTTAA GhMML10_Dt(TM-1)
------------------AAGCGTTAATTTTTATTCATTATTTACATATAAATTTTTGAACTAGTAATTTTTAAATCAGATATTTAACTGAAAATTTTAA GbMML10_Dt(3-79)
ATTGCATATAAATTATTGAAGCGTTAATTTTTATTCATTATTTACATATAAATTGTTGAACTATAAATTTTTAAATTAGATATTTAACTGAAAATTTTAA GbMML10_At(3-79)
```

```
---------------------------------------------------------------------------------------------------- GrMML10
ATTGCATATAAATTATTGAAGCGTTAATTTTTATTCATTATTTACATATAAATTGTTGAACTATAAATTTTTAAATTAGATATTTAACTGAAAATTTTAA GaMML10(Shixiya1)
```

```
         2710      2720      2730      2740      2750      2760      2770      2780
....|....|....|....|....|....|....|....|....|....|....|....|....|....|....|....|....|
CAGTATTTTTCAATTATACTAATTTCTTAATTTTCACAAACTAAAAGGACTAGCTTATTAATTTTCATAAAATAGACCATAAAAT GhMML10_At(TM-1)
CAGTATCTTTCAATTATACTAATTTCTTAATTTTCACAAACTAAAAGGACTAGCTTATTAATTTTCAGAAAATAAACCATAAAAT GhMML10_Dt(TM-1)
CAGTATCTTTCAATTATACTAATTTCTTAATTTTCACAAACTAAAAGGACTAGCTTATTAATTTTCAGAAAATAAACCATAAAAT GbMML10_Dt(3-79)
CAGTATTTTTCAATTATACTAATTTCTTAATTTTCACAAACTAAAAGGACTAGCTTATTAATTTTCATAAAATAGACCATAAAAT GbMML10_At(3-79)
------------ATTATACTAATTTCTTAATTTTCACAAACTAAAAGGACTAGCTTATTAATTTTCAGAAAATAAACCATAAAAT GrMML10
CAGTATTTTTCAATTATACTAATTTCTTAATTTTCACAAACTAAAAGGACTAGCTTATTAATTTTCATAAAATAGACCATAAAAT GaMML10(Shixiya1)
```
