# Supplementary material for: Open-Bud Duplicate Loci Are Identified as MML10s, Orthologs of MIXTA-Like Genes on Homologous Chromosomes of Allotetraploid Cotton
Source: Front Plant Sci. 2020 Feb 18;11:81. doi: 10.3389/fpls.2020.00081 (PMC7040098; doi:10.3389/fpls.2020.00081)
Supplement: Supplementary file 1 [file DataSheet_1.zip › Figure S6.pdf]

Figure S6 Alignment of the protein sequences of *MML10*. Letters with yellow background: DNA-binding motif.

```
         10        20        30        40        50        60        70        80        90       100
....|....|....|....|....|....|....|....|....|....|....|....|....|....|....|....|....|....|....|....|
---------------------------------------------MADCREKMGLKKGPWTPDEDQKLLAYIEEHGLGNWRTLPEKAGLQRCGKSCRLRW GhMML10_Dt(TM-1)
---------------------------------------------MADCREKMGLKKGPWTPDEDQKLLAYFEEHGLGNWRTLPEKAGLQRCGKSCRLRW GhMML10_At(TM-1)
-------------------------------------------MGRSPCCDKLGLKKGPWTPEEDQKLLAYIEEHGHGSWRSLPEKAGLHRCGKSCRLRW AtMYB16
-------------------------------------------MVRSPCCDKVGVKKGPWTVDEDQKLLAYIEEHGHGSWRSLPLKAGLQRCGKSCRLRW AmMYBMIXTA
MPIHVRDREKGRLQNLNRDIFCCVSPSIYQSDAKRAAFVIILIMIISPCCDKAGLKKGPWTPEEDQKLLAYIEEHGHGSWRSLPEKAGLQRCGKSCRLRW AtMYB106
---------------------------------------------MADYREKMGLKKGPWTPDEDQKLLAYFEEHGLGNWRTLPEKAGLQRCGKSCRLRW GbMML10_At(3-79)
---------------------------------------------MADYWEKMGLKKGPWTPDEDQKLLAYIEEHGLGNWRTLPEKAGFKDVERAAD--- GbMML10_Dt(3-79)
                                                                                DNA-binding motif 1

        110       120       130       140       150       160       170       180       190       200
....|....|....|....|....|....|....|....|....|....|....|....|....|....|....|....|....|....|....|....|
INYLRPDLKRGKFSLQEEQTIIQLHAFLGNRWSTIAAHLPNRTDNEIKNYWNTHVKKRFTKMGIDPTTHKPKSNHVVSPTGR----TTLNHMAQWESARL GhMML10_Dt(TM-1)
INYLRPDLKRGKFSLQEEQTIIQLHAFLGNRS-------------------------------------------------------------------- GhMML10_At(TM-1)
TNYLRPDIKRGKFNLQEEQTIIQLHALLGNRWSAIATHLPKRTDNEIKNYWNTHLKKRLVKMGIDPVTHKPKNETPLSSLGLSKNAAILSHTAQWESARL AtMYB16
ANYLRPDIKRGPFSLQEEQTIIQLHALLGNRWSAIASHLPKRTDNEIKNYWNTHLKKRLTRMGIDPVTHKPHTHNILG-HGQPKDVANLNHIAQWESARL AmMYBMIXTA
TNYLRPDIKRGKFTVQEEQTIIQLHALLGNRWSAIATHLPKRTDNEIKNYWNTHLKKRLIKMGIDPVTHKHKNETLSSSTGQSKNAATLSHMAQWESARL AtMYB106
INYLRPDLKRGKFSLQEEQTIIQLHAFLGNRWSTIAAHLPNRTDNEIKNYWNTHIKKRFTKMGIDPTTHKPKSNHVVSPTGR----TTVNHMAQWESARL GbMML10_At(3-79)
---------------------------------------------------------------------------------------------------- GbMML10_Dt(3-79)
                                   DNA-binding motif 2
```

```
       210       220       230       240       250       260       270       280       290       300
....|....|....|....|....|....|....|....|....|....|....|....|....|....|....|....|....|....|....|....|
EAEARLVKDSKNL-------PSSSSRPSPYQKSCNK-----GSKSQCLDVVKAWQSVVAGMFATSTNNSNRIIFGPDQSSGNYELDSIIPIG-------- GhMML10_Dt(TM-1)
---------------------------------------------------------------------------------------------------- GhMML10_At(TM-1)
EAEARLARESKLL---HLQHYQTKTSSQPHHHH--------GFTHKSL--LPNW---------TTKPHEDQQQLESPTSTVSFSEMKESIPA-------- AtMYB16
QAERRLVRESRLA--------QNNNKIGTIQRR--------LTWPLCLDNEQSNHYHSALLNSTSAVG-----LNQDNSFTNYSAR---PDN-------- AmMYBMIXTA
EAEARLARESKLLHLQHYQNNNNLNKSAAPQQHC-------FTQKTSTNWTKPNQG---------NGDQQ---LESPTSTVTFSENLLMPLGIPTDSSRN AtMYB106
EAEARLVKDSKKL-------PSSSSRPSPYQKSCNK-----GSKSQCLDVVKAWQSVVAGMFATSTNNSNRIIFGPDQSSGNYELDSIIPLG-------- GbMML10_At(3-79)
---------------------------------------------------------------------------------------------------- GbMML10_Dt(3-79)

       310       320       330       340       350       360       370       380       390       400
....|....|....|....|....|....|....|....|....|....|....|....|....|....|....|....|....|....|....|....|
-GNVEDELMVGNDRSKCQVPELNERFDNYMSLHDT---------------------------------THLWAAPIAENDVVEGFPDFLVHDFDYQI--- GhMML10_Dt(TM-1)
---------------------------------------------------------------------------------------------------- GhMML10_At(TM-1)
-----KIEFVGSSTGVTLMKEPEHDWINS-TMHEFETTQMG-EGIEEGFTGLLLGGDSIDRSFSGDKNETAGESSGGDCNYYEDNKNYLDSIFNFVD--- AtMYB16
-NNIYDDYEVNNIMGMIEFNNNSNYFADSLRLPGFV------EGITDISSSNIVLG--------------AGVLPSNSDNVVGYFEENWSSVLNNVASSS AmMYBMIXTA
RNNNNNESSAMIELAVSSSTSSDVSLVKEHEHDWIRQINCGSGGIGEGFTSLLIGDSVGRGLPTGKNEATAGVGNESEYNYYEDNKNYWNSILNLVD--- AtMYB106
-GNVEDELMVGNDRSKCQVPELNERSDNYMSLLDT---------------------------------THLWAAPIAENDVVESFPDFLVHDFDYQI--- GbMML10_At(3-79)
---------------------------------------------------------------------------------------------------- GbMML10_Dt(3-79)
```

```
         410
....|....|....|...
--DNEESITV--------    GhMML10_Dt(TM-1)
------------------    GhMML10_At(TM-1)
---------PSPSDSPMF    AtMYB16
SMDSPDVLVNASSSYKMY    AmMYBMIXTA
--------SSPSDSATMF    AtMYB106
--DNEESITI--------    GbMML10_At(3-79)
------------------    GbMML10_Dt(3-79)
```
