# Supplementary material for: Open-Bud Duplicate Loci Are Identified as MML10s, Orthologs of MIXTA-Like Genes on Homologous Chromosomes of Allotetraploid Cotton
Source: Front Plant Sci. 2020 Feb 18;11:81. doi: 10.3389/fpls.2020.00081 (PMC7040098; doi:10.3389/fpls.2020.00081)

Figure S7 qRT-PCR for five genes in cytokinin signal transduction pathway. Statistically significant differences were revealed using Student's t-test: *, P < 0.05. Three biological and two technical replicates were used in qRT-PCR assay. Error bars indicate the standard deviation of three biological replicates.

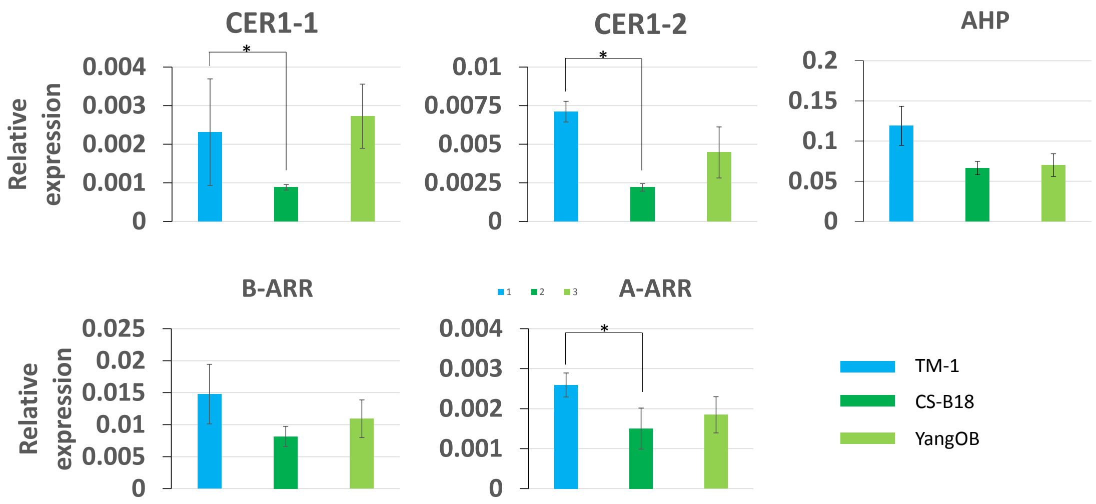

Supplement: Supplementary file 1 [file DataSheet_1.zip › Figure S7.pdf]
