# Supplementary material for: Open-Bud Duplicate Loci Are Identified as MML10s, Orthologs of MIXTA-Like Genes on Homologous Chromosomes of Allotetraploid Cotton
Source: Front Plant Sci. 2020 Feb 18;11:81. doi: 10.3389/fpls.2020.00081 (PMC7040098; doi:10.3389/fpls.2020.00081)

Figure S8 Differentially expressed genes in plant hormone signal transduction (defined by KEGG database) for the comparison of CS-B18 vs. TM-1. Green boxes indicate the down-regulated genes; red boxes indicate the up-regulated genes.

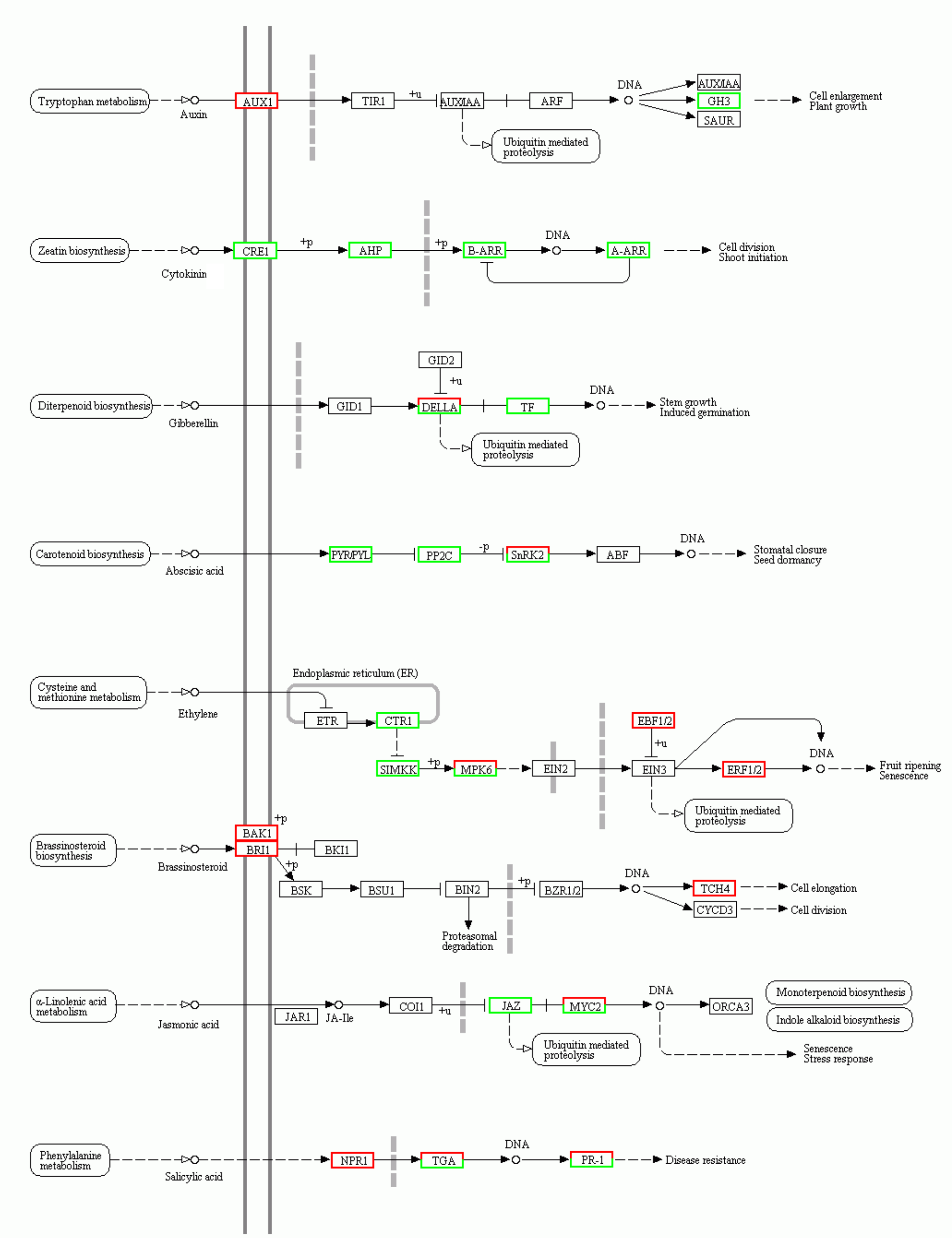

Supplement: Supplementary file 1 [file DataSheet_1.zip › Figure S8.pdf]
